# Benzene exposure and risk of lung cancer in the Norwegian Offshore Petroleum Worker cohort: a prospective case-cohort study

**DOI:** 10.1136/oemed-2023-109139

**Published:** 2023-12-28

**Authors:** Ronnie Babigumira, Marit B Veierød, H Dean Hosgood, Sven Ove Samuelsen, Magne Bråtveit, Jorunn Kirkeleit, Nathaniel Rothman, Qing Lan, Debra T Silverman, Melissa C Friesen, Nita Kaupang Shala, Tom K Grimsrud, Jo Steinson Stenehjem

**Affiliations:** 1 Department of Research, Cancer Registry of Norway, Oslo, Norway; 2 Oslo Centre for Biostatistics and Epidemiology, Department of Biostatistics, Institute of Basic Medical Sciences, University of Oslo Faculty of Medicine, Oslo, Norway; 3 Department of Epidemiology and Population Health, Albert Einstein College of Medicine, Bronx, New York, USA; 4 Department of Mathematics, University of Oslo, Oslo, Norway; 5 Department of Global Public Health and Primary Care, University of Bergen, Bergen, Norway; 6 Department of Occupational Medicine and Epidemiology, National Institute of Occupational Health (NIOH), Oslo, Norway; 7 Occupational and Environmental Epidemiology, Division of Cancer Epidemiology and Genetics, National Cancer Institute, Bethesda, Maryland, USA

**Keywords:** Epidemiology, Occupational Health, Benzene

## Abstract

**Objective:**

The objective of our study was to examine whether occupational exposure to benzene is associated with lung cancer among males in the Norwegian Offshore Petroleum Workers cohort.

**Methods:**

Among 25 347 male offshore workers employed during 1965–1998, we conducted a case-cohort study with 399 lung cancer cases diagnosed between 1999 and 2021, and 2035 non-cases sampled randomly by 5-year birth cohorts. Individual work histories were coupled to study-specific job-exposure matrices for benzene and other known lung carcinogens. Weighted Cox regression was used to estimate HRs and 95% CIs for the associations between benzene exposure and lung cancer, by major histological subtypes, adjusted for age, smoking and occupational exposure to welding fumes, asbestos and crystalline silica. Missing data were imputed.

**Results:**

For lung cancer (all subtypes combined), HRs (95% CIs) for the highest quartiles of benzene exposure versus unexposed were 1.15 (0.61 to 2.35) for cumulative exposure, 1.43 (0.76 to 2.69) for duration, and 1.22 (0.68 to 2.18) for average intensity (0.280≤P-trend≤0.741). For 152 adenocarcinoma cases, a positive trend was observed for exposure duration (P-trend=0.044).

**Conclusions:**

In this cohort of offshore petroleum workers generally exposed to low average levels of benzene, we did not find an overall clear support for an association with lung cancer (all subtypes combined), although an association was suggested for duration of benzene exposure and adenocarcinoma. The limited evidence might be due to restricted statistical power.

**WHAT IS ALREADY KNOWN ON THIS TOPIC**

- Benzene is a known human carcinogen; however, the evidence for an association between benzene and lung cancer risk remained unclear in the latest evaluation of benzene by the International Agency for Research on Cancer.

**WHAT THIS STUDY ADDS**

- This study investigated the association between occupational benzene exposure and the risk of lung cancer. Only limited evidence was found of an association between low-level benzene exposure and risk of lung cancer, suggested for adenocarcinoma and duration of benzene exposure.

**HOW THIS STUDY MIGHT AFFECT RESEARCH, PRACTICE OR POLICY**

- Risk estimates were generally non-significantly above unity, so it is important to continue monitoring benzene levels in the workplace. Our findings for adenocarcinoma motivate future prospective analyses in large datasets with adequate confounder control to better understand a possible association between benzene and lung cancer.

## Introduction

 Offshore crude oil and natural gas production has been carried out in the North Sea since the early 1970s. Benzene is a natural component of the petroleum stream and exposure in the offshore work environment may occur during drilling, production, control and maintenance of the process systems that separate crude oil, natural gas, condensate and produced water.^1 2^

In its 2017 evaluation of benzene, the International Agency for Research on Cancer (IARC) reaffirmed the classification of benzene as a group 1 carcinogen, primarily based on observational studies showing dose–response associations with lymphohaematopoietic cancers, strongly supported by mechanistic data.^3 4^ The IARC noted a dissension in the Working Group about whether a positive association between benzene and lung cancer was observed, as confounding could not be ruled out.^3^

The IARC evaluation was based on conflicting results where excesses in lung cancer incidence had been reported with varying quality of benzene exposure assessment. For lung cancer mortality, excesses have been reported among benzene-exposed workers in the UK, China and USA.^5^,^7^ We have reported an 8% overall excess of lung cancer incidence among Norwegian Offshore Petroleum Workers (NOPW) compared with the general population,^8^ while similar studies in British, Canadian and Australian petroleum workers have not.^9^,^11^ IARC highlighted the need for adequate control for potential confounding by smoking or other occupational lung carcinogens, lacking in most previous studies.^3^

After the IARC benzene evaluation, Warden *et al* reported a positive association between occupational exposure to benzene, toluene, and xylene (BTX) and lung cancer in a population-based case-control study with an expert-based retrospective exposure assessment.^12^ Recently, the SYNERGY project reported results with consistently increased risks of lung cancer according to various metrics of benzene exposure based on a large pooled population-based case-control study.^13^

In the present study, we prospectively examined the association between benzene exposure and lung cancer among males in the NOPW cohort. We used incidence data from a nationwide cancer registry, exposure information from expert-developed study-specific job-exposure matrices (JEMs) of benzene and lung carcinogens, and data on smoking history for each worker.

## Methods

### The NOPW cohort

The NOPW cohort comprises 27 917 workers engaged in offshore work for at least 20 days between 1965 and 1998. The cohort was recruited in 1998 (baseline) using a questionnaire sent to current and former offshore workers (estimated response rate 69%).^14^ The questionnaire covered work history, sociodemographic factors and lifestyle habits and has been described in detail previously.^8^

All workers in the NOPW cohort gave informed consent for a prospective follow-up.

### Study design

Each worker reported work histories for up to eight positions offshore, but work histories other than the first and last positions had to be manually extracted. The 2570 female workers were excluded as few were engaged in work with a potential for benzene exposure. Among the 25 347 male workers, we randomly drew a subcohort (n=2268 before exclusions) within strata of 5-year birth cohorts. A stratified case-cohort design allowed us to obtain complete work histories for all lung cancer cases and the subcohort with a close to negligible loss of statistical efficiency.^15^

### Follow-up and lung cancer

The cohort was linked to the Cancer Registry of Norway (CRN) and the National Population Registry using Norwegian residents' unique 11-digit personal identification number for cancer incidence, emigration and death. Reporting of incident cancer cases to the CRN has been mandatory by law since 1952, securing national data from 1953 onwards.^16^ CRN cancer data are accurate, virtually complete and timely, with verified morphology for 90.4% of the lung cancer cases.^17^ Cases were defined as first primary lung cancer (International Classification of Diseases 10th revision (ICD-10) code C34) diagnosed between 1 July 1999 and 31 December 2021 (end of follow-up). Histological subtypes of lung cancer were defined according to the ICD-Oncology 3rd revision (ICD-O-3) codes and the major subtypes were grouped into adenocarcinoma, squamous cell carcinoma and small cell carcinoma (ICD-O-3 codes in online supplemental table S1).

### Study samples

**Figure 1:**
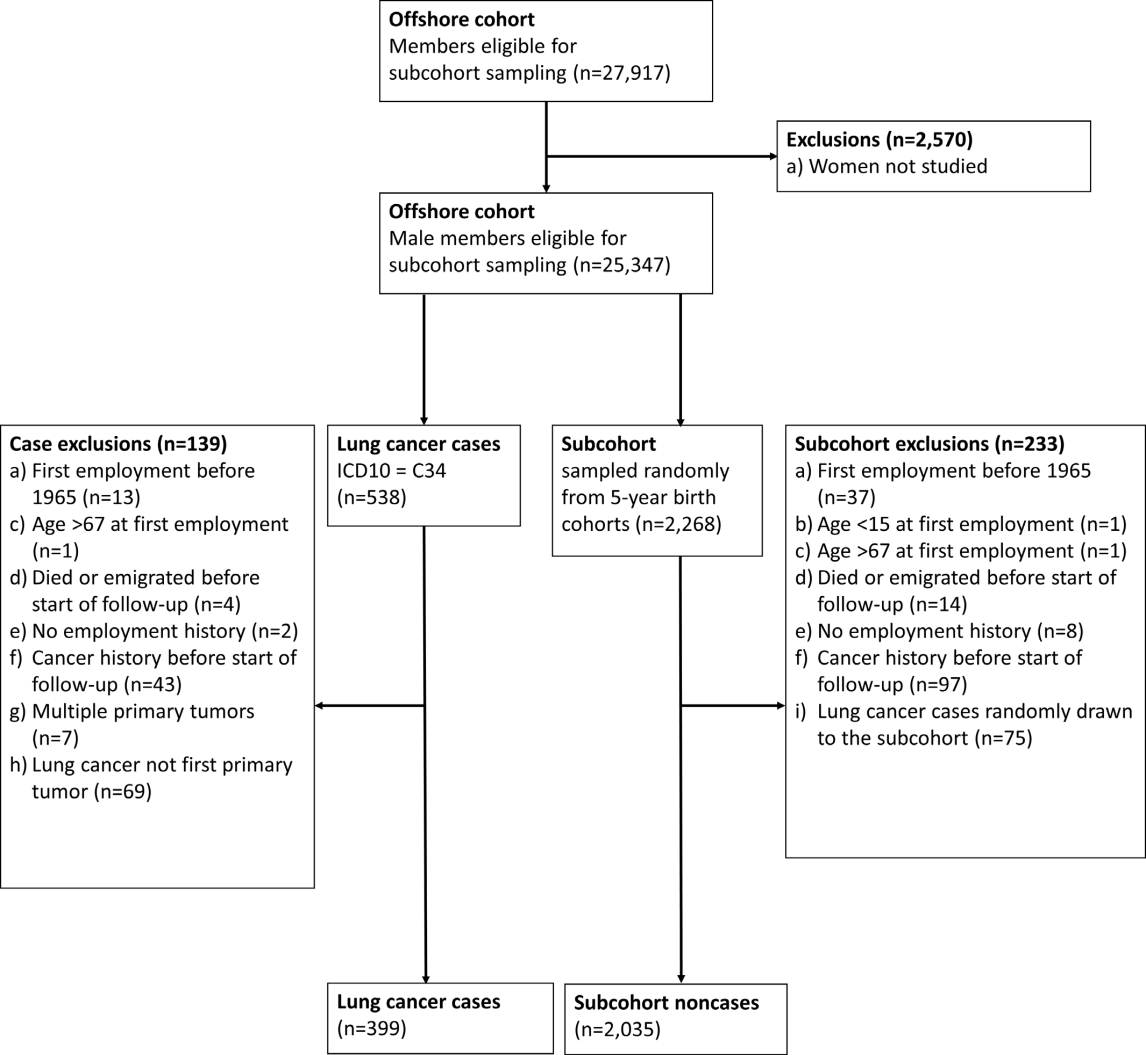
Overview of study design and exclusions. ICD-10, International Classification of Diseases 10th revision.

From the case-cohort dataset of 538 lung cancer cases and 2268 subcohort members, we excluded workers according to the criteria shown in figure 1. Lung cancer cases occurring among workers randomly drawn to the subcohort were removed from the subcohort (exclusion i) and analysed as cases only as described in Borgan *et al*.^15^ The final study sample included 399 male lung cancer cases and 2035 male subcohort non-cases. We applied the same set of exclusions for analyses of the major histological subtypes of lung cancer, restricting the cases to the subtype under study (online supplemental figures S1a–S1c).

### Exposure assessment

#### Job-exposure matrices

In 2005, a group of industrial hygienists developed expert-based JEMs using a probability-oriented approach owing to the paucity of measurement data.^18^ JEM-ratings for benzene, asbestos, crystalline silica and welding fumes were developed for 27 job categories (defined by job positions reported in the 1998 survey) and 10-year time periods (1970–1999). For each combination of agent, job category and time period, experts assessed the likelihood of exposure as unlikely=0; possible=1; probable=2 (ie, ≥50% of the job category were exposed); and probable=3 (those with the highest relative exposure among jobs with probable exposure). The assessments were based on individual and plenary interpretations of summary documents by industrial hygienists (eg, company visits/interviews, risk assessment reports, sampling reports, product data sheets). Details on the development of the probability-oriented JEMs have been published previously.^219^,^21^

In 2011, the benzene and asbestos JEMs were refined using a task-oriented approach.^18 22^ By including information from measurement data and exposure determinants on the task level rather than the job-category level, exposure estimates with greater contrasts between job categories and time periods were obtained. Benzene measurement data were used to assist in scoring determinants for the exposure intensity of the relevant tasks. Subsequently, task duration and frequency were combined to create a semiquantitative benzene exposure burden score specific to job category and time period. The relative contribution from dermal absorption and inhalation was not taken into account in the refined benzene JEM. The semiquantitative ratings of benzene exposure were translated into proportional corresponding estimates of parts per million (ppm) on the basis of the full shift mean benzene exposure for process operators offshore estimated from 204 personal benzene measurements collected in the period 1994 to 2003.^18 23^ Individual work-history data (start, stop, job category) for up to eight employments per worker between 1965 and 1998 were linked to the JEMs. Overlapping employment records were handled by collapsing jobs within the same category and splitting jobs of different categories into proportionally equal parts, according to a previously described method.^24^

Exposure duration was defined as years exposed to each agent. For all agents, cumulative exposure estimates were derived by multiplying the JEM ratings by days of duration within each year and then by summing the products from start of first employment until either the end of last employment, or 31 December 1998. Average exposure intensities were derived by dividing cumulative exposure by exposure duration. For benzene exposure metrics cumulative, duration and average intensity, the workers were divided into quartiles among exposed workers, and yielding five categories (unexposed and quartiles 1–4). For sensitivity analyses, we extrapolated benzene exposure data for those still employed and exposed in 1998. We assigned the last reported benzene exposure intensity to each year during follow-up, that is, from 1998 until the retirement year (age 67 years), death, emigration, or end of follow-up (2021), whichever came first.

#### Covariates

The study participants reported daily smoking (yes, no) and the daily number of cigarettes or pipes of tobacco (0, 1–4, 5–9, 10–14, 15–19, 20–24, 25–29, ≥30) for each age span (15–19, 20–29, 30–39, 40–49, 50–59, ≥60). From these data, we derived individual smoking status at baseline (never, former, current), duration of smoking (years), smoking pack years (years*packs per day (1 pack=20 cigarettes)), and average intensity (defined as pack years/duration for all current smokers at baseline). From status and pack years at baseline, we computed a four-category smoking variable: never/former<15 years duration, former with ≥15 years duration, current low (<median of average intensity) and current high (≥median of average intensity). Education was recorded in the questionnaire as compulsory, vocational, folk high school and upper secondary (the latter two collapsed into upper secondary), and university/college. We did not address potential confounding from benzene exposures outside offshore work as we previously have found that such occupations had no effect on lymphohematopoietic cancers.^23^

### Data analysis

Missing data patterns were examined, and we used multiple imputation by chained equations to impute missing observations for smoking (duration and pack years) and education, assuming missing at random. The imputation model included all covariates and case status. The covariates had up to 4% missing; hence, we imputed eight datasets. Weighted Cox proportional hazards regression, adapted to the case-cohort design,^15^ was used to estimate HRs and 95% CIs for the associations between lung cancer and smoking metrics and benzene exposure (unexposed, cumulative, duration, intensity, lagged exposure) on each of the imputed datasets. These results were then combined using Rubin’s combination rules.^25^

We used directed acyclic graphs (DAGs)^26^ to arrive at three models. In model 1, we adjusted for age only (as the time scale). In model 2, we additionally adjusted for smoking (online supplemental figure S2). In model 3, we further adjusted for exposure to welding fumes, asbestos and crystalline silica (online supplemental figure S3). Although diesel exhaust exposure levels in the Norwegian offshore working environment have been reported to be relatively low,^27 28^ we performed additional analysis with adjustment also for diesel exhaust exposure (online supplemental model 4, figure S4). We also examined the diesel exhaust–lung cancer association for cumulative, duration and average intensity metrics of diesel exhaust, adjusted for age and smoking, and estimated Spearman rank correlation coefficients, r_sp_, between continuous variables of benzene, crystalline silica, diesel exhaust, welding fumes and asbestos exposure.

We conducted analyses stratified by start of first employment (<1980, ≥1980) to explore whether technical and safety improvements on the Norwegian continental shelf during the 1980s altered the results.^2^

To explore potential latency of a benzene-lung cancer association, we conducted analyses with time-varying benzene exposure.^29^ To assess the impact of early exposure, we modelled lagged benzene exposure (10, 15, or 20 years). We also analysed the most recent exposure within 5-year, 10-year or 15-year windows from the start to the end of follow-up, using extrapolated data during follow-up. Finally, we conducted a traditional time-dependent exposure analysis based on extrapolated data during follow-up.

We examined the association between employment duration and lung cancer risk to explore the potential role of a healthy worker survivor effect (HWSE), defined as a continuing selection process where those who remain employed tend to be healthier than those who left employment.^30^

We tested for trend across exposure categories using the median exposure within each level of the exposure metric. The proportionality assumption was checked using Schoenfeld residuals, log–log plots and Kaplan-Meier survival curves and found satisfactory. Data analyses were performed using Stata V.17.1.^31^

## Results

Nearly half of the 399 cases and 2035 non-cases were born in 1940–1949, and mean age at start of follow-up was 51.5 and 53.9 years, respectively (table 1). Non-cases had a higher prevalence of university/college education (19.0%) than the cases (9.5%). There were more current smokers, and fewer never and former smokers among the cases compared with the non-cases. Cases had higher means of pack years and of smoking duration. There were minor differences between cases and non-cases in the year of first employment, and a higher proportion of cases than non-cases worked in maintenance and catering/administration.

**Table 1:** Baseline characteristics of the case-cohort study sample in the Norwegian Offshore Petroleum Workers cohort

Variables	Cases (n=399)	Non-cases (n=2035)
**Sociodemographic**		
Birth cohort, n (%)		
1910–1919	0 (0.0)	2 (0.1)
1920–1929	6 (1.5)	146 (7.2)
1930–1939	70 (17.5)	475 (23.3)
1940–1949	194 (48.6)	933 (45.8)
1950–1959	103 (25.8)	345 (17.0)
1960–1969	24 (6.0)	117 (5.7)
1970–1979	2 (0.5)	17 (0.8)
Age at start of follow-up (years), mean (SD)	51.5 (8.4)	53.9 (9.7)
Education, n (%)		
Compulsory	102 (25.6)	319 (15.7)
Vocational	192 (48.1)	1031 (50.7)
Upper secondary	65 (16.3)	280 (13.8)
University/college	38 (9.5)	386 (19.0)
Missing	2 (0.5)	19 (0.9)
Smoking status, n (%)		
Never	4 (1.0)	453 (22.3)
Former	107 (26.8)	845 (41.5)
Current	277 (69.4)	667 (32.8)
Missing	11 (2.8)	70 (3.4)
Smoking pack years, mean (SD)*	26.4 (13.8)	15.4 (14.7)
Smoking duration, mean (SD)*	33.5 (12.3)	22.1 (16.9)
**Occupational**		
Employment duration (years), mean (SD)†	12.2 (7.5)	12.1 (7.4)
Year of first employment, n (%)		
1965–1969	18 (4.5)	58 (2.9)
1970–1974	40 (10.0)	233 (11.4)
1975–1979	150 (37.6)	772 (37.9)
1980–1984	88 (22.1)	432 (21.2)
1985–1989	62 (15.5)	336 (16.5)
1990–1994	28 (7.0)	136 (6.7)
1995–1998	13 (3.3)	68 (3.3)
Main activity last position, n (%)		
Production	29 (7.3)	178 (8.7)
Drilling	29 (7.3)	181 (8.9)
Maintenance	226 (56.6)	1037 (51.0)
Catering/office/administration	64 (16.0)	288 (14.2)
Miscellaneous	46 (11.5)	326 (16.0)
Missing	5 (1.3)	25 (1.2)

* Missing in continuous variables: smoking pack years (n=81), smoking duration (n=81).

† Duration calculated using complete work history data.

The smoking-lung cancer analysis (table 2), yielded age-adjusted HRs (95% CIs) of 12 (6.81 to 20) for current smokers with average intensity<median (13 g/day), and 20 (12 to 34) for current smokers with average intensity≥median, compared with those with 0–15 year duration. The estimated HRs were consistently elevated among smokers with a strong and monotonic dose response for the major histological subtypes of lung cancer.

**Table 2:** HRs with 95% CIs for lung cancer and major subtypes according to smoking variables among males in the Norwegian Offshore Petroleum Workers (NOPW) cohort, 1999–2021

	All lung cancers(n=399)	Adenocarcinoma(n=152)	Squamous cell carcinoma(n=88)	Small cell carcinoma(n=62)
	HR* (95% CI)	HR* (95% CI)	HR* (95% CI)	HR* (95% CI)
Smoking†				
Never/Former (0–15 years duration)	1.00 (reference)	1.00 (reference)	1.00 (reference)	NA‡
Former (≥15 years duration)	6.25 (3.64 to 11)	7.51 (3.15 to 18)	4.02 (1.33 to 12)	1.00 (reference)
Current (avg. int.<median avg. int.)§	12 (6.81 to 20)	13 (5.60 to 31)	9.91 (3.40 to 29)	4.24 (2.09 to 8.60)
Current (avg. int.≥median avg. int.)§	20 (12 to 34)	18 (7.83 to 43)	23 (8.00 to 63)	8.58 (4.46 to 17)

* Adjusted for age (as the time scale).

† Variable constructed by splitting former smokers into </≥15 years duration and current smokers into </≥median average intensity (0.65). Missing values (n=81) were imputed.

‡ Not applicable (no cases).

§ Median=13 g of tobacco per day.

avg. int.average intensityyrsyears

Models 1, 2 and 3 showed similar results in the analyses of benzene exposure and lung cancer risk (table 3), and model 3 results are presented. The HR estimates for all exposure metrics and lung cancer were non-significantly close to or above unity with no indication of a trend (P-trend=0.631, 0.280 and 0.741 for cumulative, duration and intensity, respectively).

**Table 3:** HRs with 95% CIs of lung cancer according to benzene exposure among males in the Norwegian Offshore Petroleum Workers cohort, 1999–2021

Benzene metric	Cases/non-cases	Model 1*	Model 2†	**Model3** **‡**
		HR§ (95% CI)	HR§ (95% CI)	HR§ (95% CI)
Cumulative (ppm years)				
Unexposed	112/655	1.00 (reference)	1.00 (reference)	1.00 (reference)
Q1 (0.000−<0.019)	85/332	1.36 (1.00 to 1.85)	1.34 (0.96 to 1.86)	1.47 (0.90 to 2.40)
Q2 (0.019−<0.071)	72/345	1.26 (0.92 to 1.75)	1.27 (0.90 to 1.79)	1.30 (0.75 to 2.26)
Q3 (0.071−<0.175)	63/354	1.09 (0.78 to 1.52)	1.07 (0.75 to 1.51)	1.18 (0.64 to 2.15)
Q4 (0.176–0.879)	67/349	1.19 (0.86 to 1.64)	1.17 (0.84 to 1.65)	1.15 (0.61 to 2.16)
*P-trend*		*0.815*	*0.859*	*0.631*
Duration (years)				
Unexposed	112/655	1.00 (reference)	1.00 (reference)	1.00 (reference)
Q1 (1−4)	69/382	0.99 (0.72 to 1.37)	1.01 (0.72 to 1.43)	1.14 (0.67 to 1.95)
Q2 (5−10)	85/366	1.41 (1.04 to 1.92)	1.36 (0.99 to 1.89)	1.55 (0.93 to 2.61)
Q3 (11−16)	64/301	1.24 (0.89 to 1.74)	1.26 (0.88 to 1.79)	1.56 (0.90 to 2.70)
Q4 (17−34)	69/331	1.33 (0.96 to 1.84)	1.26 (0.89 to 1.77)	1.43 (0.76 to 2.69)
*P-trend*		*0.035*	*0.095*	*0.280*
Average intensity (ppm)				
Unexposed	112/655	1.00 (reference)	1.00 (reference)	1.00 (reference)
Q1 (0.000−<0.004)	90/327	1.47 (1.08 to 2.00)	1.36 (0.98 to 1.88)	1.42 (0.87 to 2.30)
Q2 (0.004−<0.007)	66/351	1.13 (0.82 to 1.57)	1.21 (0.86 to 1.71)	1.41 (0.80 to 2.49)
Q3 (0.007−<0.014)	73/344	1.31 (0.95 to 1.80)	1.19 (0.85 to 1.67)	1.37 (0.77 to 2.46)
Q4 (0.014–0.046)	58/358	1.00 (0.71 to 1.40)	1.07 (0.75 to 1.52)	1.22 (0.68 to 2.18)
*P-trend*		*0.485*	*0.871*	*0.741*

* Adjusted for age (as the time scale).

† Adjusted for age (as the time scale), smoking.

‡ Adjusted for age (as the time scale), smoking, welding fumes, asbestos and crystalline silica

§ Missing values in covariates were imputed

Qquartile

No significant trends were found for benzene exposure and squamous cell carcinoma or small cell carcinoma (0.156≤P-trend≤0.914) (table 4). A significant positive trend was found for duration of benzene exposure and adenocarcinoma (P-trend=0.044), the histological subgroup with the highest number of cases. The highest HR was found in the upmost quartile (HR=2.02, 95% CI 0.80 to 5.11).

**Table 4:** HRs with 95% CIs of the major histological subtypes of lung cancer according to benzene exposure among males in the Norwegian Offshore Petroleum Workers (NOPW) cohort, 1999–2021

Benzene metric	Adenocarcinoma (n=152)		Squamous cell carcinoma (n=88)		Small cell carcinoma (n=62)	
	C/NC	Model 3*	C/NC	Model 3*	C/NC	Model 3*
		HR† (95% CI)		HR† (95% CI)		HR† (95% CI)
Cumulative (ppm years)						
Unexposed	45/665	1.00 (reference)	27/671	1.00 (reference)	14/676	1.00 (reference)
Q1 (0.000–<0.019)	30/341	1.25 (0.60 to 2.61)	19/343	1.03 (0.40 to 2.69)	13/346	1.11 (0.35 to 3.49)
Q2 (0.019–<0.071)	20/355	1.01 (0.44 to 2.29)	21/353	0.73 (0.25 to 2.08)	13/357	1.07 (0.32 to 3.56)
Q3 (0.071–<0.175)	35/359	2.20 (0.98 to 4.90)	5/364	0.19 (0.05 to 0.74)	11/363	0.83 (0.19 to 3.57)
Q4 (0.176–0.879)	22/363	1.27 (0.50 to 3.28)	16/358	0.39 (0.10 to 1.56)	11/362	1.19 (0.32 to 4.33)
*P-trend*		*0.986*		*0.283*		*0.642*
Duration (years)						
Unexposed	45/665	1.00 (reference)	27/671	1.00 (reference)	14/676	1.00 (reference)
Q1 (1–4)	24/389	0.93 (0.43 to 2.03)	12/392	0.66 (0.20 to 2.17)	12/391	1.11 (0.32 to 3.79)
Q2 (5–10)	28/375	1.39 (0.62 to 3.11)	21/374	1.11 (0.42 to 2.94)	14/379	1.04 (0.33 to 3.27)
Q3 (11–16)	26/313	1.82 (0.80 to 4.17)	11/313	0.83 (0.30 to 2.35)	10/314	1.23 (0.36 to 4.21)
Q4 (17–34)	29/341	2.02 (0.80 to 5.11)	17/339	0.97 (0.24 to 3.90)	12/344	1.32 (0.32 to 5.40)
*P-trend*		*0.044*		*0.914*		*0.639*
Average intensity (ppm)						
Unexposed	45/665	1.00 (reference)	27/671	1.00 (reference)	14/676	1.00 (reference)
Q1 (0.000–<0.004)	28/337	1.15 (0.56 to 2.38)	22/338	1.00 (0.40 to 2.51)	15/344	1.18 (0.40 to 3.50)
Q2 (0.004–<0.007)	30/362	1.84 (0.81 to 4.17)	13/360	0.73 (0.23 to 2.31)	12/362	1.27 (0.35 to 4.54)
Q3 (0.007–<0.014)	27/353	1.65 (0.70 to 3.91)	17/354	0.66 (0.21 to 2.02)	15/355	1.68 (0.49 to 5.78)
Q4 (0.014–0.046)	22/366	1.34 (0.55 to 3.27)	9/366	0.50 (0.16 to 1.61)	6/367	0.72 (0.19 to 2.79)
*P-trend*		*0.883*		*0.156*		*0.323*

* Adjusted for age (as the time scale), smoking, welding fumes, asbestos and crystalline silica

† Missing values were imputed

CcasesNCnon casesQquartile

The results of the analyses with additional adjustment for diesel exhaust exposure (online supplemental table S2) were similar to the model 3 results. Moderate correlation was found between benzene and diesel exhaust exposure metrics (online supplemental figure S5 and table S9): r_sp_=0.41 for (cumulative), r_sp_=0.51 for (duration) and r_sp_=0.33 (average intensity). No association was found between diesel exhaust and lung cancer for any of the diesel exhaust metrics (0.416≤P-trend≤0. 427, model 2, online supplemental table S3), supporting the above analyses with model 3 as the main model.

Analysis stratified by year of first employment before or after 1980 (online supplemental tables S4a and S4b, Model 3) showed similar results, although HRs for Q3 and Q4 were elevated among those starting before 1980.

Estimates generally decreased with increasing lag period for lung cancer overall according to cumulative and duration exposure of benzene (online supplemental table S5a, Model 3) with no statistically significant trends (0.121≤P-trend≤0.953). Lagged analyses by histological subtype did not reveal any clear patterns (online supplemental table S5b). For adenocarcinoma, estimates were largest in the 10-year lag while for squamous cell carcinoma, estimates were mostly below unity.

We examined the effect of recent benzene exposure in 5-year, 10-year and 15-year windows before observation (online supplemental table S6, Model 3). In all windows, Q1 were consistently higher than Q4, but with some fluctuations in the middle cateogories. For the 15-year windows, Q1 were elevated for both cumulative (HR=1.60, 95% CI 1.10 to 2.33) and average intensity (HR=1.21, 95% CI 0.79 to 1.87) of exposure.

We analysed benzene exposure as a time-varying covariate with no lags or windows, using extrapolated data during follow-up for workers still active in 1998 (online supplemental table S7, Model 3). The overall results showed slightly increased estimates in Q1, Q2 and Q3, but with a drop in Q4 for cumulative exposure and exposure duration.

Lung cancer risk according to employment duration (online supplemental table S8), showed no clear pattern with lung overall or small cell carcinoma, but showed a suggestive upward trend for adenocarcinoma and a downward trend for small cell carcinoma.

There were moderate correlations between benzene and other occupational coexposures cumulative (0.0376≤r_sp_≤0.6608), duration (0.2420≤r_sp_≤0.8026) and average intensity (−0.0504≤r_sp_≤0.6175) (online supplemental table S9).

## Discussion

In this large prospective cohort study, with cancer incidence data, industry-specific expert-derived JEMs, and detailed smoking history, we only found limited evidence for an association between low-level exposure to benzene (<0.050 avg. ppm and<1 ppm yrs) and lung cancer (all subtypes combined), although the estimates in the preferred DAG-based model with no lagged exposure or exposure windows, were generally above unity. Exposure duration seemed to yield a more dose–response like pattern than cumulative and average intensity of exposure, and an association was suggested for adenocarcinoma with exposure duration, although no clear pattern was seen for the other histological subtypes. Sensitivity analyses of time of first employment, lagged exposure, most recent exposure and time-varying exposure did not add much to the main findings.

A key component of the IARC 2017 evaluation was the studies conducted in the large historical cohort of benzene-exposed workers in China.^6 32^ In the first follow-up (1972–1987), a 70% increase in lung cancer mortality was reported among those exposed to ≥400 ppm years compared with the unexposed.^6^ In the second follow-up (1972–1999), this persisted, where Linet *et al* reported a 50% significantly increased lung cancer mortality among benzene-exposed workers.^32^ However, in the latest follow-up, participants were classified as either ‘ever exposed’ or ‘never exposed’ based on whether the factory they worked in used benzene.^32^ Further, smoking data were lacking, which may have contributed to the increased lung cancer mortality. These limitations and the higher benzene exposure levels in the Chinese cohort, which covered a wide range of industries across over 600 factories, may explain the differences between these results and the present study.

In a population-based case-control study of lung cancer in Montreal, lung cancer was associated with exposure to benzene after adjustment for smoking.^12^ The authors noted that their results suggested that exposure to benzene, toluene or xylene (each agent assessed separately) were associated with modest increases in lung cancer risk and that smoking did not clearly modify the effects of BTX exposures.^12^

Results from the SYNERGY project^13^ showed that risks of lung cancer increased consistently for all histological subtypes and for different metrics of occupational benzene exposure. Their analyses were adjusted for age, sex, smoking and other known occupational lung carcinogens. Wan *et al* reported a decline in risk with increasing time since last exposure,^13^ while we were not able to confirm this pattern by looking only at the most recent exposure. Similar to our results, adenocarcinoma increased with increasing exposure duration. Compared with 0.879 ppm years for quartile 4 seen in our data, Wan *et al* reported >5 ppm years in their top category. The lower exposure levels in the NOPW cohort, compared with SYNERGY, and the substantially lower number of cases may explain the lack of a more consistent exposure–risk pattern seen in our data.

The studies conducted in China, Montreal and by the SYNERGY project all reported elevated risks of lung cancer associated with occupational benzene exposure.^12 13 32^ However, these studies had higher exposure levels and/or more lung cancer cases/deaths than we observed in our cohort. The effect size of the association between benzene and lung cancer is suggested to be moderate, which may hamper the possibility to observe risk patterns consistent with those seen in the large studies.

Benzene is an established leukemogen and known to have toxic effects on blood and bone marrow found at lower levels than earlier expected.^33^ Accordingly, we have previously observed an association between benzene and lymphohaematopoietic cancers in the NOPW cohort.^23^ Further, benzene has been shown to be genotoxic,^34^ and to be associated with alterations to telomere length.^35^ Also, increased telomerase activity has been shown in fibroblast-like human LL24 lung cell lines exposed to benzene, but not in human alveolar epithelial adenocarcinoma.^36^ A biological explanation for the increased risk estimates of adenocarcinoma observed in our study is therefore difficult to find, but the systemic effect of benzene may be more important than direct epithelial contact through inhalation, as it seems to be when intraperitoneal injection of benzene in male rodents has been found to induce lung adenomas.^37^

Strengths of the NOPW cohort include extensive information on work history and potential confounding factors; industry-specific JEMs developed for the NOPW cohort, which have proven useful in detecting an association between benzene and lymphohaematopoietic cancers; the prospective case-cohort study design that precludes differential recall bias between cases and non-cases; and linkage to a nationwide cancer registry with high validity. In addition, socioeconomic status (SES) is relatively homogenous within the cohort, which reduces the potential for confounding by SES as opposed to population-based studies where several countries, industries and occupational groups contribute. We conducted a rigorous set of sensitivity analyses, but they did not materially differ from our main results.

Limitations include exposure misclassification resulting from self-reporting of work histories, some of which started 30 years before baseline in 1998, although reporting of work histories has been found to be robust.^38^ The stronger association seen for the duration metric, compared with the cumulative metric, may also be due to misclassifications in the JEM-ratings. Also, the lack of exposure data during follow-up, may have resulted in exposure misclassification, as partly seen in our sensitivity analysis with extrapolated data during follow-up where estimates for Q4 slightly dropped compared with the main analysis. As the NOPW cohort was relatively young in 1998 and included only those who were alive, lung cancer occurring during the first three decades of petroleum activity was not covered, and we cannot rule out bias from the HWSE due to left truncation (delayed entry). However, our analyses of lagged exposure duration showed that estimates in Q4 increased slightly with increasing lag time, supporting a weak HWSE. Furthermore, estimates for overall lung cancer dropped slightly with employment duration, suggesting a weak HWSE.

In this cohort of offshore petroleum workers with low average exposure levels to benzene, we found generally non-significantly increased risks of lung cancer, except for duration of benzene exposure and adenocarcinoma, the largest histological subgroup. The moderate risk levels seen in other studies suggest that our study has limitations linked to statistical power.

## supplementary material

10.1136/oemed-2023-109139 online supplemental file 1

## Data Availability

Data may be obtained from a third party and are not publicly available. The data that support the findings of this study are available from the CRN (cohort data and cancer data) and the National Population Register (death and emigration data) but restrictions apply to the availability of these data, which were used under license for the current study, and so are not publicly available. Requests for data sharing/case pooling for projects with necessary approvals and legal basis according to the EU General Data Protection Regulation (GDPR) may be directed to principal investigator Dr Jo S Stenehjem; email: jo.stenehjem@kreftregisteret.no.
