# Supplementary material for: Benzene exposure and risk of lung cancer in the Norwegian Offshore Petroleum Worker cohort: a prospective case-cohort study
Source: Occup Environ Med. 2023 Dec 28;81(1):e109139. doi: 10.1136/oemed-2023-109139 (PMC11881047; doi:10.1136/oemed-2023-109139)
Supplement: online supplemental file 1 [file oemed-81-1-s001.pdf]

SUPPLEMENTAL MATERIAL

Content

- Table S1: Number of lung cancer cases by histological subtype
- Overview of study designs and exclusions by subtype
    - Figure S1a: Adenocarcinoma
    - Figure S1b: Squamous cell carcinoma
    - Figure S1c: Small cell carcinoma
- Directed acyclic graphs (DAGs)
    - Figure S2: DAG of Model 2
    - Figure S3: DAG of Model 3
    - Figure S4: DAG of Model 4
- Diesel exhaust exposure as a potential confounder – Model 4
    - Table S2: Model 4
    - Figure S5: Correlation between diesel exhaust and benzene.
    - Tables S3: Diesel exhaust exposure and lung cancer risk
    - Discussion of diesel exhaust as a potential confounder
- Analysis stratified by year of first employment
    - Table S4a: <1980
    - Table S4b: ≥1980
- Latency analyses with time-varying benzene exposure
    - Table S5a: Lagged analysis
    - Table S5b: Lagged analysis by histological subtype
    - Table S6: Most recent benzene exposure
    - Table S7: Time-varying benzene exposure during follow-up
- Table S8: Lung cancer risk according to employment duration
- Table S9. Spearman rank correlation coefficients (r_{sp}) for occupational co-exposures

Number of lung cancer cases by histological subtype

Table S1. Number of lung cancer cases by histological subtype among 25,347 males in the Norwegian Offshore Petroleum Workers (NOPW) cohort followed 1999–2021.

Cancer type	Number of cases
Lung (all cases)	399
Non-small cell carcinoma	337
Adenocarcinoma[1]	152
Squamous cell carcinoma[2]	88
Large cell carcinoma[3*]	9
Large cell neuroendocrine carcinoma[4*]	8
Non-small cell carcinoma UNS[5*]	44
Carcinoid[6*]	6
Other[7*]	9
Unknown[8*]	21
Small cell carcinoma[9]	62

[1-9]Codes from the International Classification of Diseases Oncology 3rd revision (ICD-O-3) used to classify histological subtypes of lung cancer.

[1]7032, 7033, 7039, 7508, 8140, 8148, 8211, 8230, 8250, 8251, 8252, 8253, 8254, 8255, 8256, 8257, 8260, 8263, 8265, 8310, 8323, 8333, 8410, 8460, 8470, 8480, 8481, 8490, 8550, 8551, 8570, 8574, 8576, 8940

[2]8052, 8070, 8071, 8072, 8073, 8074, 8075, 8076, 8077, 8082

[3]8012

[4]8013

[5]7160,7161,8010,8020,8046;

[6]8240,8241,8243,8245,8249 ;

[7]8022, 8030, 8031, 8032, 8033, 8040, 8083, 8123, 8140, 8200, 8244, 8246, 8250, 8430, 8560, 8562, 8973, 8980,

[8]8000,8001

[9]8041

*Not analysed separately

Overview of study designs and exclusions by subtype

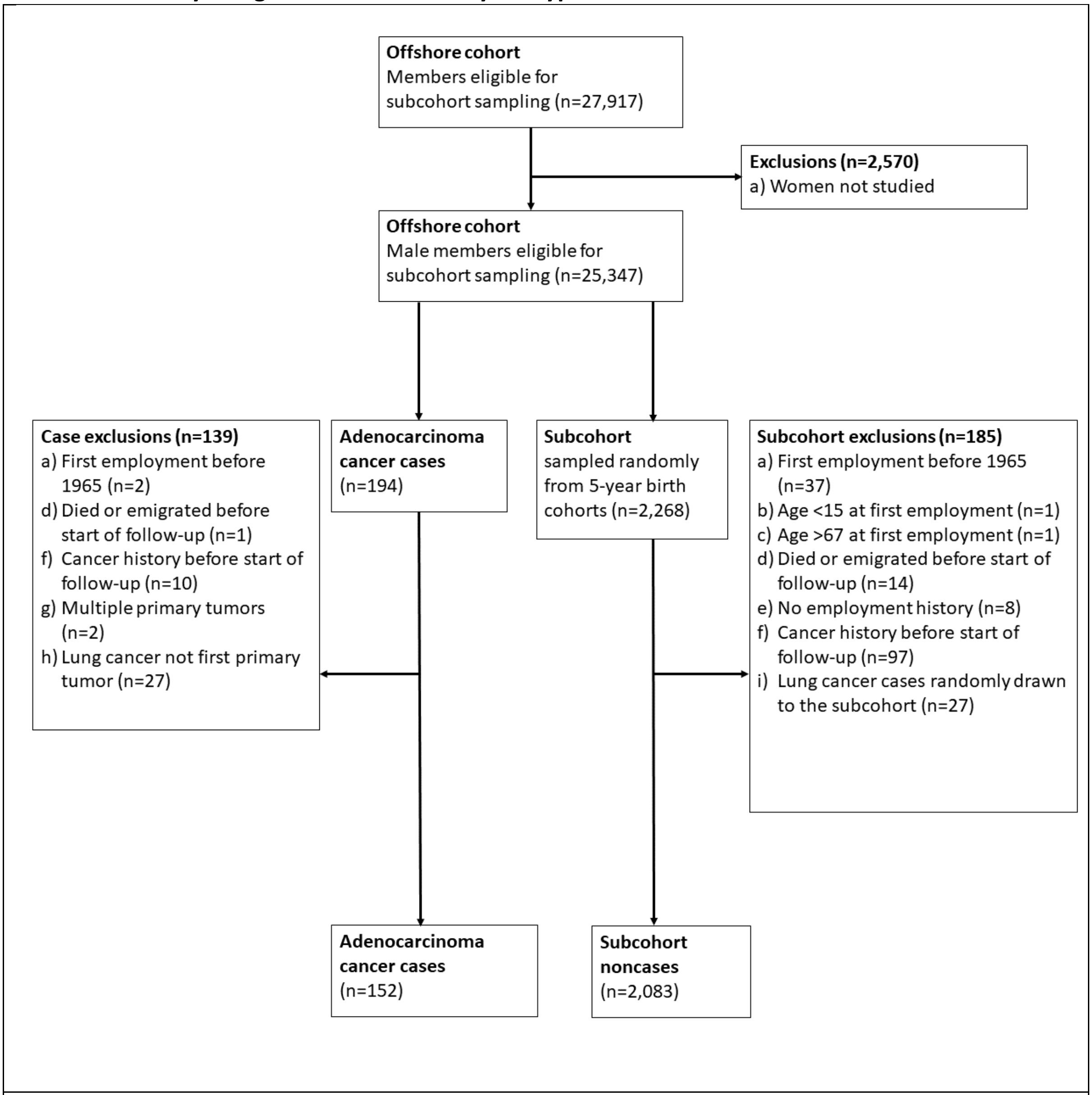

Figure S1a. Overview of study design and exclusions for the analyses of adenocarcinoma.

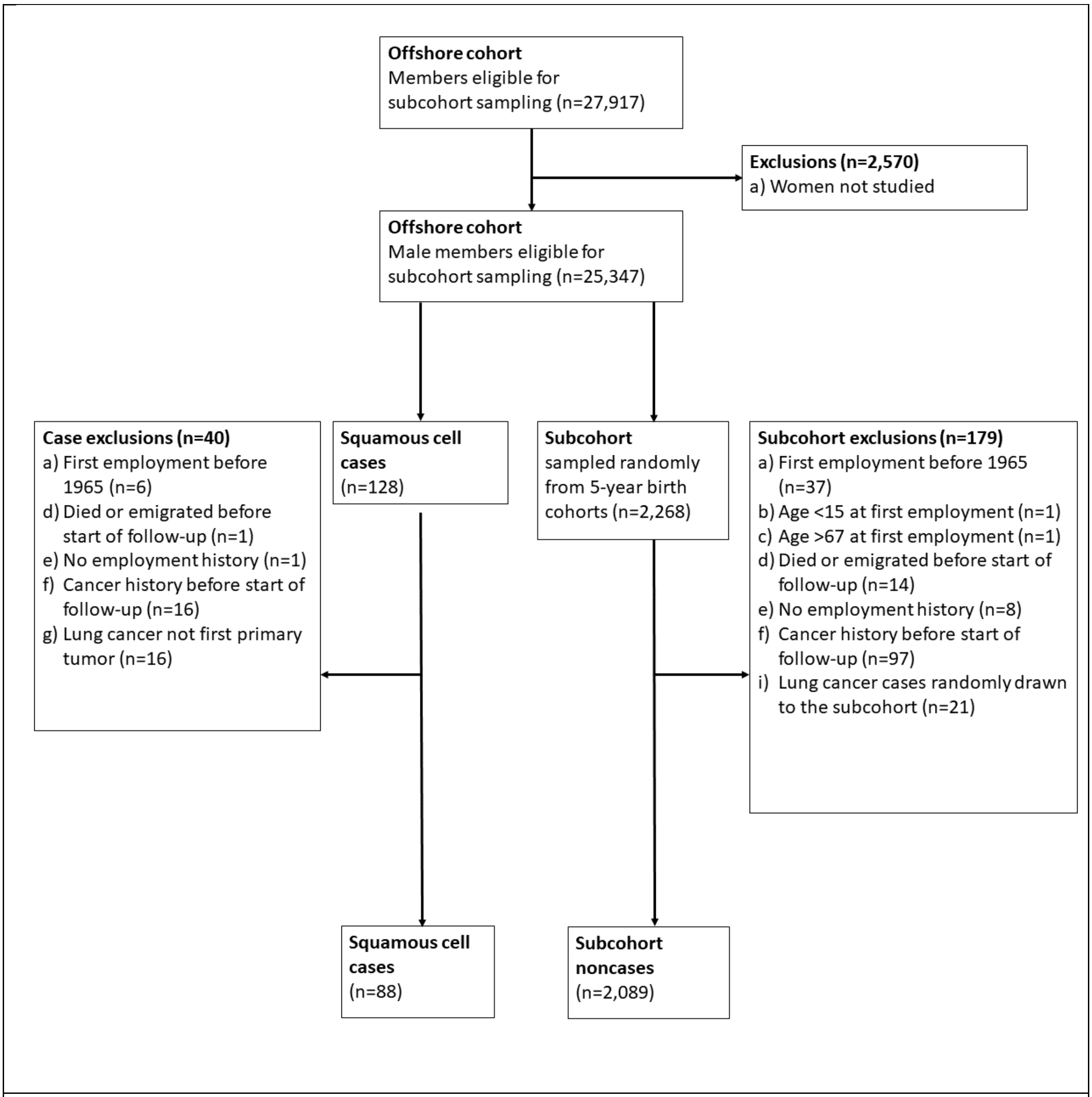

Figure S1b. Overview of study design and exclusions for the analyses of squamous cell carcinoma.

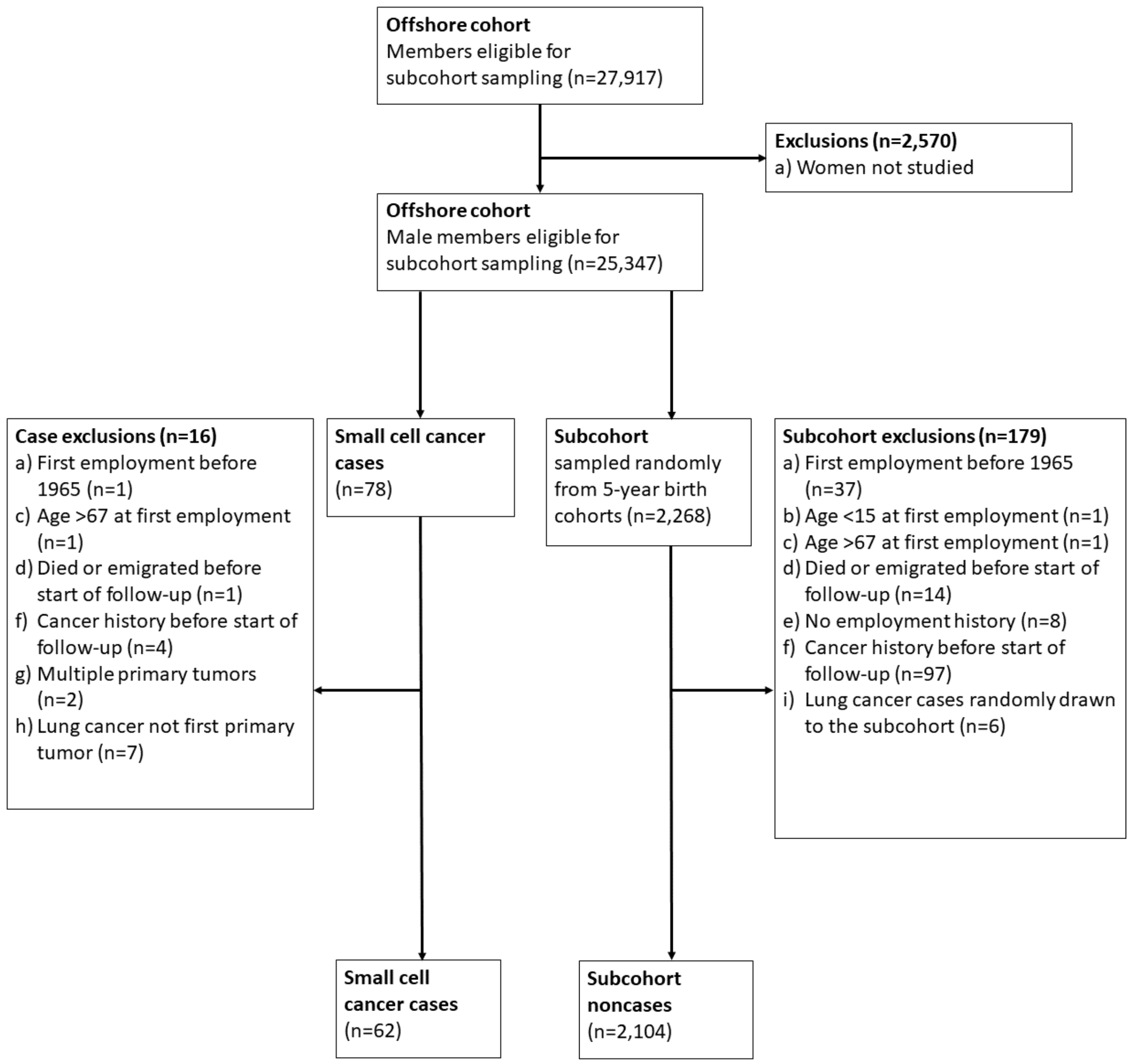

Figure S1c. Overview of study design and exclusions for the analyses of small cell carcinoma.

Directed acyclic graphs (DAGs)

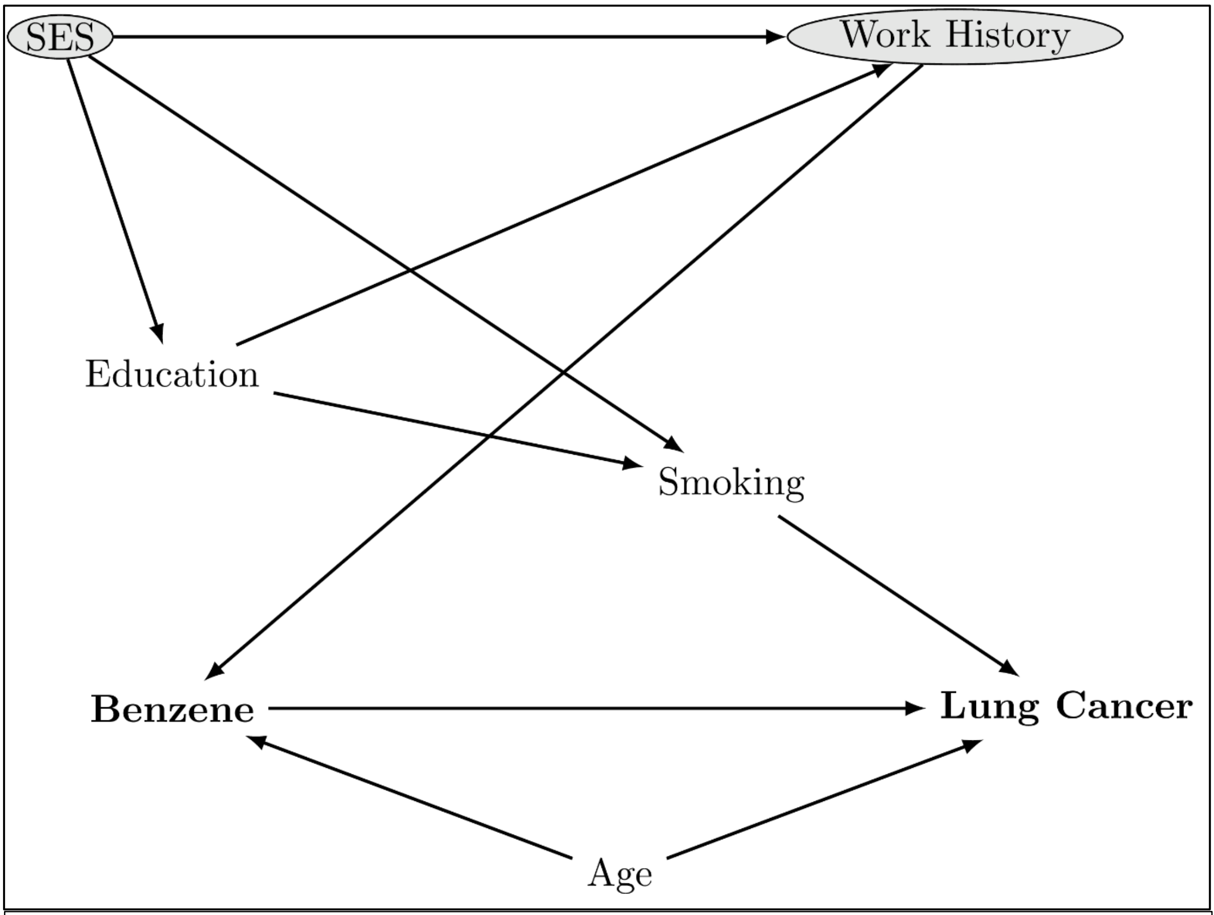

Figure S2. Directed acyclic graph showing our assumptions about causal pathways between occupational benzene (exposure of interest, limited to offshore-related activities), other factors, and lung cancer (endpoint). Socio-economic status (SES) and lifetime work history (work history) are unobserved/latent variables. All nodes represent baseline covariate levels. We assumed the following paths:

- benzene → lung cancer
- benzene ←age[1] →lung cancer
- work history → benzene → lung cancer
- SES[2,3] → work history[4] → benzene → lung cancer
- work history ← education[2] → smoking[1,5] → lung cancer
- SES → education → smoking → lung cancer

Based on these assumptions, we need to adjust for smoking and age to obtain the total effect of occupational benzene exposure on lung cancer.

[1]Bade BC, Dela Cruz CS. Lung Cancer 2020: Epidemiology, Etiology, and Prevention. Clinics in Chest Medicine. 2020;41(1):1-24.
[2]Larsen IK, Myklebust TÅ, Babigumira R, Vinberg E, Møller B, Ursin G. Education, income and risk of cancer: results from a Norwegian registry-based study. Acta Oncologica. 2020;59(11):1300-7
[3]Hovanec J, Siemiatycki J, Conway DI, Olsson A, Stücker I, Guida F, et al. Lung cancer and socioeconomic status in a pooled analysis of case-control studies. PLOS ONE. 2018;13(2):e0192999.
[4]Weissman DN, Howard J. Work-Related Lung Cancer: The Practitioner's Perspective. Am J Public Health. 2018;108(10):1290-2.
[5]Wild CP, Weiderpass E, Stewart BW, editors (2020). World Cancer Report: Cancer Research for Cancer Prevention. Lyon, France: International Agency for Research on Cancer. Available from: http://publications.iarc.fr/586. Licence: CC BY-NC-ND 3.0 IGO.

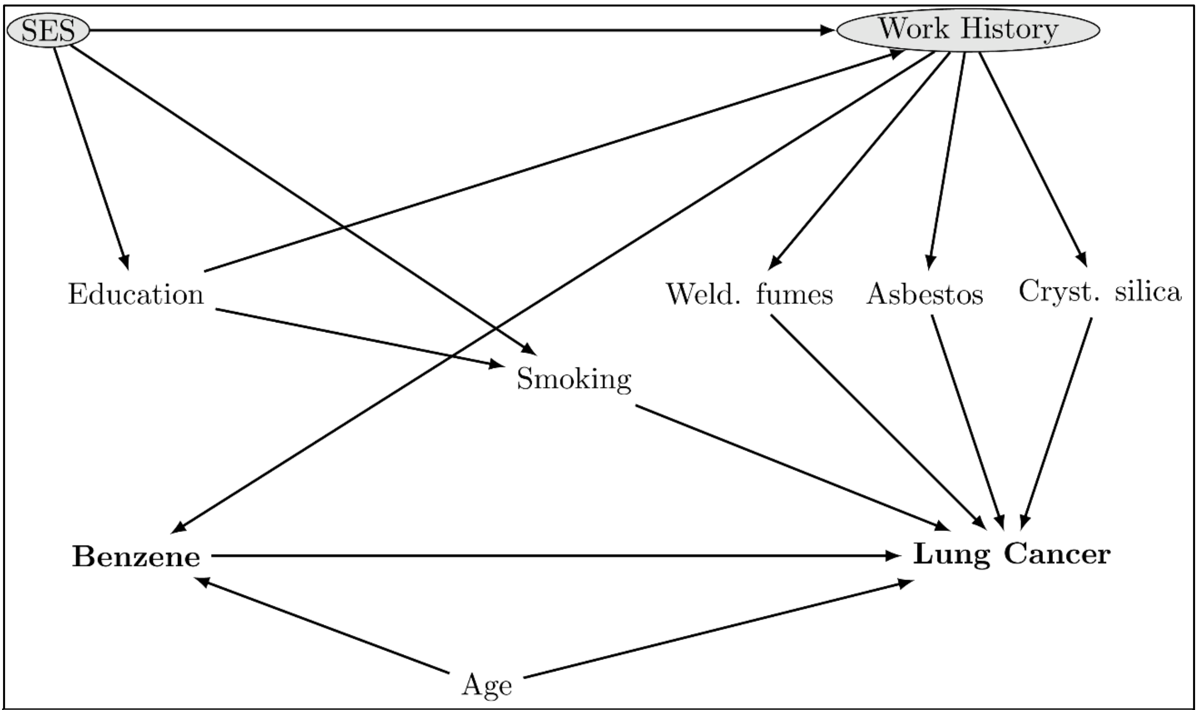

Figure S3. Directed acyclic graph showing our assumptions about causal pathways between occupational benzene (exposure of interest, limited to offshore-related activities), other factors and lung cancer (endpoint). Socio-economic status (SES) and lifetime work history (work history) are unobserved/latent variables. All nodes represent baseline covariate levels. We assumed the following paths;

- benzene → lung cancer
- benzene ←age[1] →lung cancer
- work history → benzene → lung cancer
- SES[2,3] → work history[4] → benzene → lung cancer
- work history ← education[2] → smoking[1,5] → lung cancer
- SES → education → smoking → lung cancer
- work history → welding fumes[6] → lung cancer
- work history → asbestos[5,6] → lung cancer
- work history → crystalline silica[5,6] → lung cancer

Based on these assumptions, we need to adjust for smoking, age, welding fumes, asbestos, and crystalline silica to obtain the total effect of occupational benzene exposure on lung cancer.

[1]Bade BC, Dela Cruz CS. Lung Cancer 2020: Epidemiology, Etiology, and Prevention. Clinics in Chest Medicine. 2020;41(1):1-24.
[2]Larsen IK, Myklebust TÅ, Babigumira R, Vinberg E, Møller B, Ursin G. Education, income and risk of cancer: results from a Norwegian registry-based study. Acta Oncologica. 2020;59(11):1300-7
[3]Hovanec J, Siemiatycki J, Conway DI, Olsson A, Stücker I, Guida F, et al. Lung cancer and socioeconomic status in a pooled analysis of case-control studies. PLOS ONE. 2018;13(2):e0192999.
[4]Weissman DN, Howard J. Work-Related Lung Cancer: The Practitioner's Perspective. Am J Public Health. 2018;108(10):1290-2.
[5]Wild CP, Weiderpass E, Stewart BW, editors (2020). World Cancer Report: Cancer Research for Cancer Prevention. Lyon, France: International Agency for Research on Cancer. Available from: http://publications.iarc.fr/586. Licence: CC BY-NC-ND 3.0 IGO.
[6] IARC. List of classifications by cancer sites with sufficient or limited evidence in humans, IARC Monograhs Volumes 1–1332023 26.06.2023. Available from: https://monographs.iarc.who.int/wp-content/uploads/2019/07/Classifications_by_cancer_site.pdf.

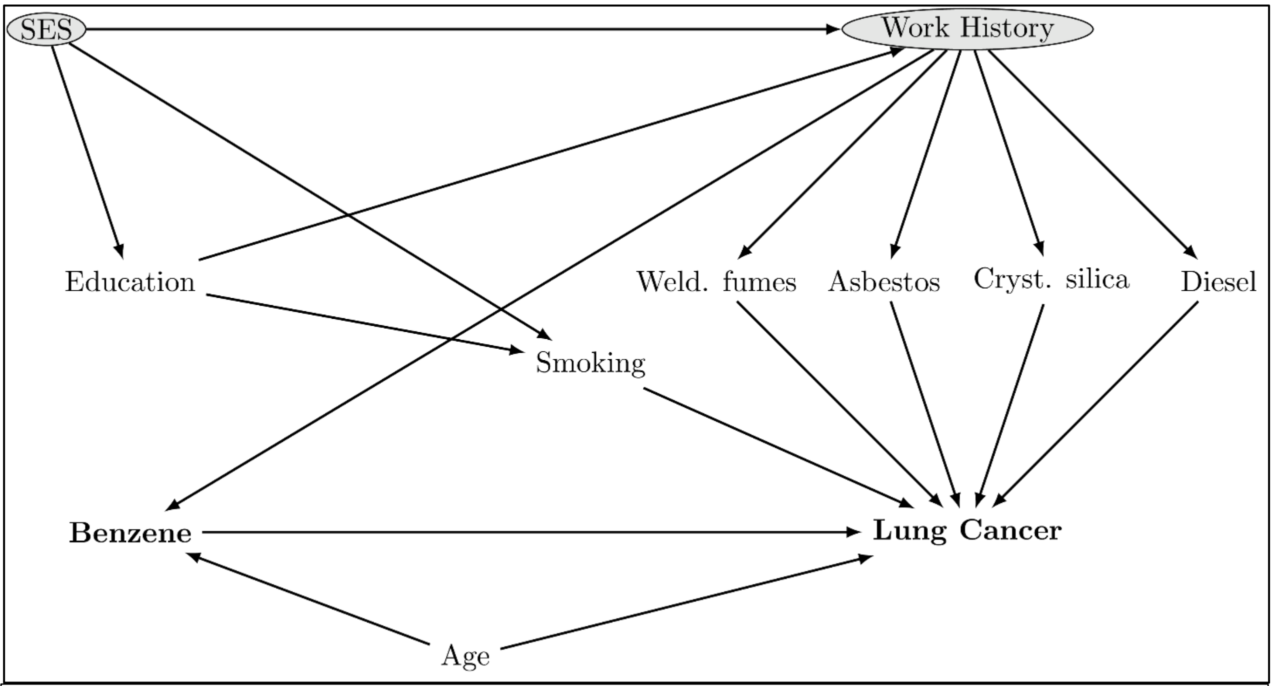

Figure S4. Directed acyclic graph showing our assumptions about causal pathways between occupational benzene (exposure of interest, limited to offshore-related activities), other factors and lung cancer (endpoint). Socio-economic status (SES) and lifetime work history (work history) are unobserved/latent variables. All nodes represent baseline covariate levels. We assumed the following paths;

- benzene → lung cancer
- benzene ←age[1] →lung cancer
- work history → benzene → lung cancer
- SES[2,3] → work history[4] → benzene → lung cancer
- work history ← education[2] → smoking[1,5] → lung cancer
- SES → education → smoking → lung cancer
- work history → welding fumes[6] → lung cancer
- work history → asbestos[5,6] → lung cancer
- work history → crystalline silica[5,6] → lung cancer
- work history → diesel exhaust[5,6] → lung cancer

Based on these assumptions, we need to adjust for smoking, age, welding fumes, asbestos, crystalline silica and diesel exhaust to obtain the total effect of occupational benzene exposure on lung cancer.

[1]Bade BC, Dela Cruz CS. Lung Cancer 2020: Epidemiology, Etiology, and Prevention. Clinics in Chest Medicine. 2020;41(1):1-24.
[2]Larsen IK, Myklebust TÅ, Babigumira R, Vinberg E, Møller B, Ursin G. Education, income and risk of cancer: results from a Norwegian registry-based study. Acta Oncologica. 2020;59(11):1300-7
[3]Hovanec J, Siemiatycki J, Conway DI, Olsson A, Stücker I, Guida F, et al. Lung cancer and socioeconomic status in a pooled analysis of case-control studies. PLOS ONE. 2018;13(2):e0192999.
[4]Weissman DN, Howard J. Work-Related Lung Cancer: The Practitioner's Perspective. Am J Public Health. 2018;108(10):1290-2.
[5]Wild CP, Weiderpass E, Stewart BW, editors (2020). World Cancer Report: Cancer Research for Cancer Prevention. Lyon, France: International Agency for Research on Cancer. Available from: http://publications.iarc.fr/586. Licence: CC BY-NC-ND 3.0 IGO.
[6] IARC. List of classifications by cancer sites with sufficient or limited evidence in humans, IARC Monograhs Volumes 1–1332023 26.06.2023. Available from: https://monographs.iarc.who.int/wp-content/uploads/2019/07/Classifications_by_cancer_site.pdf

Diesel exhaust exposure as a potential confounder

Table S2, Figure S5, and Table S3 are presented below. Table S2 shows the results of analyses using Model 4, in which diesel exhaust was added to Model 3 as a potential confounder (DAG in Figure S4). Scatter plots and correlation coefficients between diesel exhaust and benzene exposure metrics are presented in Figure S5. Table S3 presents the results of diesel exhaust and lung cancer risk analyses.

Table S2, Model 4. Hazard ratios (HR) with 95% confidence intervals (CIs) of all lung and the major histological subtypes of lung cancer according to benzene exposure among males in the Norwegian Offshore Petroleum Workers (NOPW) cohort, 1999–2021.

		**All lung[a] (n=399)**	**Adenocarcinoma[a] (n=152)**	**Squamous cell carcinoma[a] (n=88)**	**Small cell carcinoma[a] (n=62)**
**Benzene metric**	**C/NC**	**HR[b] (95% CI)**	**HR[b] (95% CI)**	**HR[b] (95% CI)**	**HR[b] (95% CI)**
Cumulative (ppm-years)					
Unexposed	112/655	1.00 (reference)	1.00 (reference)	1.00 (reference)	1.00 (reference)
Q1 (0.000 - <0.019)	85/332	1.65 (0.98, 2.77)	1.46 (0.66, 3.23)	1.05 (0.39, 2.83)	1.27 (0.36, 4.42)
Q2 (0.019 - <0.071)	72/345	1.39 (0.79, 2.44)	1.08 (0.45, 2.56)	0.68 (0.23, 1.99)	1.29 (0.36, 4.62)
Q3 (0.071 - <0.175)	63/354	1.28 (0.69, 2.40)	2.49 (1.04, 5.95)	0.17 (0.04, 0.66)	0.99 (0.22, 4.45)
Q4 (0.176-0.879)	67/349	1.27 (0.67, 2.38)	1.46 (0.56, 3.80)	0.39 (0.10, 1.56)	1.31 (0.35, 4.90)
P-Trend		*0.693*	*0.904*	*0.299*	*0.726*
Duration (years)					
Unexposed	112/655	1.00 (reference)	1.00 (reference)	1.00 (reference)	1.00 (reference)
Q1 (1 - 4)	69/382	1.24 (0.71, 2.17)	0.99 (0.44, 2.24)	0.62 (0.19, 1.98)	1.35 (0.37, 4.97)
Q2 (5 - 10)	85/366	1.67 (0.98, 2.87)	1.50 (0.64, 3.50)	1.11 (0.41, 3.02)	1.17 (0.34, 4.08)
Q3 (11 - 16)	64/301	1.73 (0.98, 3.05)	2.12 (0.88, 5.09)	0.85 (0.29, 2.47)	1.32 (0.35, 4.96)
Q4 (17 - 34)	69/331	1.57 (0.83, 2.97)	2.28 (0.88, 5.90)	1.01 (0.26, 3.91)	1.50 (0.36, 6.26)
P-Trend		*0.202*	*0.026*	*0.818*	*0.653*
Average intensity (ppm)					
Unexposed	112/655	1.00 (reference)	1.00 (reference)	1.00 (reference)	1.00 (reference)
Q1 (0.000 - <0.004)	90/327	1.57 (0.95, 2.59)	1.36 (0.63, 2.94)	1.00 (0.39, 2.56)	1.36 (0.43, 4.37)
Q2 (0.004 - <0.007)	66/351	1.66 (0.90, 3.06)	2.22 (0.92, 5.35)	0.69 (0.21, 2.23)	1.92 (0.46, 7.94)
Q3 (0.007 - <0.014)	73/344	1.67 (0.90, 3.10)	2.15 (0.87, 5.28)	0.70 (0.22, 2.22)	2.22 (0.58, 8.47)
Q4 (0.014 - 0.046)	58/358	1.30 (0.72, 2.34)	1.43 (0.57, 3.57)	0.47 (0.15, 1.47)	0.87 (0.22, 3.38)
P-Trend		*0.648*	*0.988*	*0.103*	*0.385*

Abbreviations: C=cases, NC= non cases, Q=quartile

[a]Adjusted for age (as the time scale), smoking, welding fumes, asbestos, crystalline silica, and diesel exhaust

[b]Missing values were imputed

Cumulative exposure

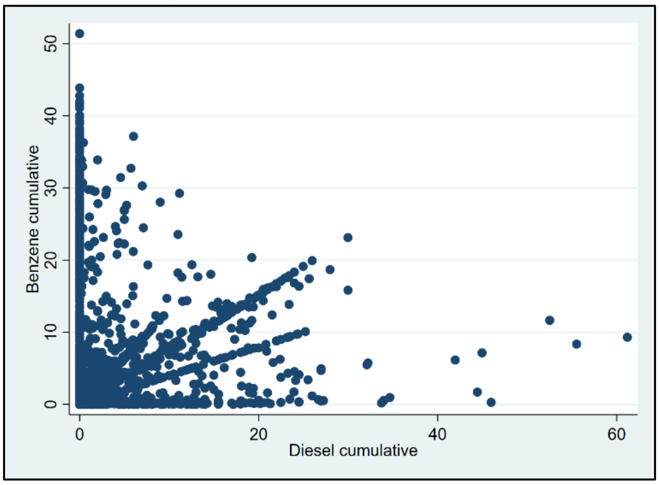

Spearman correlation coefficient=0.41

Duration

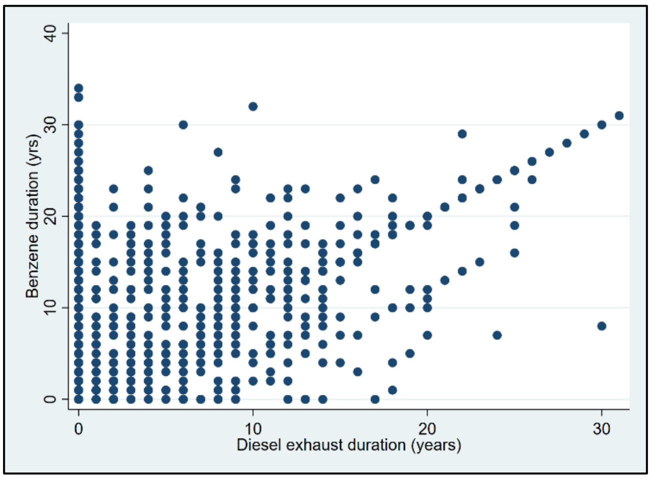

Spearman correlation coefficient=0.51

Average intenstiy

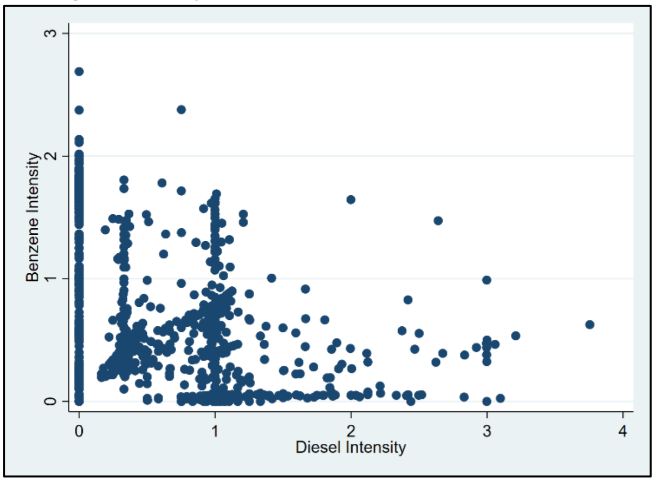

Spearman correlation coefficient=0.33

Figure S5. Correlation between benzene and diesel exhaust metrics

Examination of the association between diesel exhaust exposure and lung cancer risk

Table S3. Hazard ratios (HR) with 95% confidence intervals (CIs) of lung cancer according to diesel exhaust exposure among males in the Norwegian Offshore Petroleum Workers cohort, 1999–2021.

		**Model D1[a]**	**Model D2[b]**
**Diesel exhaust metric**	**Cases/Non-cases**	**HR[c] (95% CI)[c]**	**HR[c] (95% CI)**
**Cumulative**			
Unexposed	219/1098	1.00 (reference)	1.00 (reference)
Q1 (0.165 - <1.895)	41/239	0.86 (0.60, 1.23)	0.96 (0.65, 1.41)
Q2 (1.977 - <4.579)	38/241	0.85 (0.59, 1.24)	0.88 (0.59, 1.30)
Q3 (4.614 - <10.489)	51/228	1.14 (0.82, 1.60)	0.99 (0.69, 1.42)
Q4 (10.493 - 61.181)	50/229	1.00 (0.72, 1.40)	0.89 (0.62, 1.26)
P-trend		*0.722*	*0.422*
**Duration (years)**			
Unexposed	219/1098	1.00 (reference)	1.00 (reference)
Q1 (1 - 3)	48/262	0.91 (0.64, 1.27)	0.98 (0.68, 1.42)
Q2 (4 - 7)	46/218	1.09 (0.77, 1.55)	0.99 (0.68, 1.43)
Q3 (8 - 13)	41/226	0.91 (0.63, 1.31)	0.86 (0.58, 1.26)
Q4 (14 - 31)	45/231	0.97 (0.68, 1.37)	0.89 (0.62, 1.29)
P-trend		*0.842*	*0.427*
**Average intensity**			
Unexposed	219/1098	1.00 (reference)	1.00 (reference)
Q1 (0.165 - <0.332)	39/241	0.85 (0.59, 1.23)	0.94 (0.63, 1.41)
Q2 (0.332 - <0.940)	44/235	0.95 (0.67, 1.35)	0.98 (0.68, 1.42)
Q3 (0.941 - <1.000)	56/223	1.25 (0.90, 1.73)	1.11 (0.78, 1.57)
Q4 (1.000 - 4.004)	41/238	0.83 (0.58, 1.18)	0.72 (0.49, 1.05)
P-trend		*0.932*	*0.416*

Abbreviations: D=diesel exhaust; Q=quartile

[a]Adjusted for age (as the time scale).

[b]Adjusted for age (as the time scale) and smoking.

[c]Missing values in smoking were imputed.

Evaluation of diesel exhaust as a potential confounder of the benzene–lung cancer association

In Table S2 with Model 4, we found that HRs consistently increased by 10% compared to Model 3. This increase is likely due to negative bias or negative confounding (Szklo and Janiver-Nieto, 2000) since we (A) observed positive correlations between diesel exhaust and benzene metrics, and (B) in Table S3, Model D2, observed a null or negative association between diesel exhaust and lung cancer. The lack of an association between diesel exhaust and lung cancer in our data may be due to low concentrations in the offshore working environment. Concentrations have been reported by the Norwegian Institute of Occupational Health to be low (Solbu et al., 2012).

References

- Solbu K, Bakke B, Friisk G, Skaugset NP. Dieseleksos i arbeidsatmosfæren i norsk olje- og gassindustri – Dagens eksponeringsbilde. Report in Norwegian. Oslo, Norway: STAMI. No 4 (13), 2012. URL: https://stami.no/content/uploads/2015/03/STAMI-rapport-nr-4-2012.pdf
- Szklo M, Javier-Nieto F. Identifying non causal associations: Confounding.In: Epidemiology: Beyond the Basics. Gaithersburg, MD: Aspen Publishers,Inc.; 2000:203.

Analysis stratified by year of first employment.

Before 1980

Table S4a. Hazard ratios (HR) with 95% confidence intervals (CIs) of lung cancer according to benzene exposure among males in the Norwegian Offshore Petroleum Workers cohort whose first employment was before 1980, 1999–2021.

		**Model 1[a]**	**Model 2[b]**	**Model 3[c]**
**Benzene metric**	**Cases/Non-cases**	**HR[d] (95% CI)**	**HR[d] (95% CI)**	**HR[d] (95% CI)**
Cumulative (ppm-years)				
Unexposed	54/317	1.00 (reference)	1.00 (reference)	1.00 (reference)
Q1 (0.000 - <0.025)	43/182	1.22 (0.79, 1.88)	1.19 (0.75, 1.89)	1.39 (0.66, 2.91)
Q2 (0.025 - <0.091)	36/189	1.28 (0.81, 2.00)	1.35 (0.84, 2.17)	1.62 (0.74, 3.55)
Q3 (0.092 - <0.198)	36/189	1.06 (0.67, 1.66)	1.12 (0.70, 1.80)	1.49 (0.68, 3.29)
Q4 (0.199 - 0.879)	38/186	1.20 (0.77, 1.87)	1.12 (0.71, 1.78)	1.50 (0.64, 3.52)
P-Trend		*0.705*	*0.925*	*0.823*
Duration (years)				
Unexposed	54/317	1.00 (reference)	1.00 (reference)	1.00 (reference)
Q1 (1 - 5)	36/206	0.98 (0.63, 1.54)	1.02 (0.63, 1.65)	1.10 (0.48, 2.51)
Q2 (6 - 12)	39/181	1.43 (0.91, 2.24)	1.54 (0.97, 2.46)	1.69 (0.81, 3.55)
Q3 (13 - 20)	46/212	1.25 (0.82, 1.91)	1.19 (0.77, 1.85)	1.54 (0.73, 3.26)
Q4 (21 - 34)	32/147	1.12 (0.70, 1.78)	1.08 (0.66, 1.77)	1.55 (0.63, 3.79)
P-Trend		*0.301*	*0.510*	*0.224*
Average intensity (ppm)				
Unexposed	54/317	1.00 (reference)	1.00 (reference)	1.00 (reference)
Q1 (0.000 - <0.004)	45/180	1.23 (0.80, 1.89)	1.12 (0.71, 1.77)	1.36 (0.66, 2.79)
Q2 (0.004 - <0.008)	37/189	1.18 (0.76, 1.85)	1.31 (0.82, 2.10)	1.70 (0.78, 3.71)
Q3 (0.008 - <0.013)	40/184	1.28 (0.83, 1.97)	1.26 (0.80, 1.98)	1.73 (0.77, 3.88)
Q4 (0.013 - 0.046)	31/193	1.03 (0.65, 1.65)	1.08 (0.67, 1.76)	1.35 (0.58, 3.14)
P-Trend		*0.943*	*0.760*	*0.992*

Abbreviations: Q=quartile

[a]Adjusted for age (as the time scale).

[b]Adjusted for age (as the time scale) and smoking.

[c]Adjusted for age (as the time scale), smoking, welding fumes, asbestos and crystalline silica.

[d]Missing values in covariates were imputed.

After or in 1980

Table S4b. Hazard ratios (HR) with 95% confidence intervals (CIs) of lung cancer according to benzene exposure among males in the Norwegian Offshore Petroleum Workers cohort whose first employment was after 1980, 1999–2021.				
		**Model 1[a]**	**Model 2[b]**	**Model 3[c]**
**Benzene metric**	**Cases/Non-cases**	**HR[d] (95% CI)**	**HR[d] (95% CI)**	**HR[d] (95% CI)**
Cumulative (ppm-years)				
Unexposed	58/338	1.00 (reference)	1.00 (reference)	1.00 (reference)
Q1 (0.000 - <0.016)	42/150	1.48 (0.95, 2.29)	1.50 (0.93, 2.42)	1.93 (0.97, 3.85)
Q2 (0.017 - <0.053)	38/154	1.39 (0.89, 2.19)	1.34 (0.83, 2.17)	1.76 (0.84, 3.67)
Q3 (0.053 - <0.128)	25/167	0.97 (0.58, 1.61)	0.98 (0.57, 1.69)	0.94 (0.40, 2.23)
Q4 (0.129 - 0.555)	29/163	1.04 (0.64, 1.70)	1.03 (0.61, 1.73)	0.71 (0.28, 1.83)
P-Trend		*0.571*	*0.565*	*0.070*
Duration (years)				
Unexposed	58/338	1.00 (reference)	1.00 (reference)	1.00 (reference)
Q1 (1 - 3)	33/163	1.09 (0.68, 1.74)	1.18 (0.71, 1.95)	1.78 (0.83, 3.83)
Q2 (4 - 7)	39/151	1.52 (0.97, 2.39)	1.33 (0.82, 2.15)	1.88 (0.91, 3.88)
Q3 (8 - 12)	36/156	1.33 (0.84, 2.11)	1.29 (0.79, 2.12)	1.40 (0.66, 3.01)
Q4 (13 - 19)	26/164	0.99 (0.60, 1.63)	1.06 (0.62, 1.80)	0.90 (0.37, 2.17)
P-Trend		*0.612*	*0.613*	*0.648*
Average intensity (ppm)				
Unexposed	58/338	1.00 (reference)	1.00 (reference)	1.00 (reference)
Q1 (0.000 - <0.004)	45/147	1.66 (1.07, 2.56)	1.63 (1.02, 2.61)	1.84 (0.95, 3.56)
Q2 (0.004 - <0.007)	32/160	1.22 (0.76, 1.95)	1.30 (0.78, 2.16)	1.51 (0.67, 3.36)
Q3 (0.007 - <0.015)	31/161	1.13 (0.70, 1.82)	0.94 (0.56, 1.57)	1.01 (0.42, 2.42)
Q4 (0.015 - 0.041)	26/166	0.91 (0.55, 1.51)	1.05 (0.62, 1.78)	0.99 (0.42, 2.32)
P-Trend		*0.322*	*0.645*	*0.308*
Abbreviations: Q=quartile				
[a]Adjusted for age (as the time scale).				
[b]Adjusted for age (as the time scale) and smoking.				
[c]Adjusted for age (as the time scale), smoking, welding fumes, asbestos and crystalline silica.				
[d]Missing values in covariates were imputed.				

Latency analyses with time-varying benzene exposure

Lagged analysis

Table S5a. Hazard Ratios (HR) of lung cancer according to lagged benzene exposure among males in the Norwegian Offshore Petroleum Workers (NOPW) cohort, 1999–2021.

			Model 1[a]	Model 2[b]	Model 3[c]
**Benzene metric**	**Cases**	**Person years**	**HR[d] (95% CI)**	**HR[d] (95% CI)**	**HR[d] (95% CI)**
Cumulative (ppm-years)					
10-year lag					
Unexposed	117	13876	1.00 (reference)	1.00 (reference)	1.00 (reference)
Q1 (0.000 - <0.017)	81	6802	1.36 (1.00, 1.86)	1.33 (0.95, 1.85)	1.38 (0.86, 2.22)
Q2 (0.017 - <0.065)	70	6768	1.26 (0.91, 1.74)	1.28 (0.91, 1.80)	1.26 (0.74, 2.14)
Q3 (0.065 - <0.157)	65	6784	1.13 (0.82, 1.57)	1.11 (0.78, 1.56)	1.09 (0.61, 1.96)
Q4 (0.157 - 0.879)	66	6831	1.07 (0.77, 1.48)	1.05 (0.75, 1.47)	0.93 (0.50, 1.71)
P-trend			*0.719*	*0.668*	*0.212*
15-year lag					
Unexposed	126	15259	1.00 (reference)	1.00 (reference)	1.00 (reference)
Q1 (0.000 - <0.015)	78	6456	1.33 (0.98, 1.82)	1.29 (0.93, 1.79)	1.22 (0.78, 1.91)
Q2 (0.015 - <0.057)	66	6399	1.21 (0.88, 1.67)	1.26 (0.90, 1.77)	1.13 (0.68, 1.88)
Q3 (0.057 - <0.137)	61	6450	1.04 (0.75, 1.45)	1.03 (0.73, 1.44)	0.84 (0.48, 1.47)
Q4 (0.137 - 0.879)	68	6497	1.01 (0.74, 1.39)	0.99 (0.71, 1.38)	0.77 (0.43, 1.39)
P-trend			*0.509*	*0.461*	*0.121*
20-year lag					
Unexposed	141	18146	1.00 (reference)	1.00 (reference)	1.00 (reference)
Q1 (0.000 - <0.012)	79	5695	1.51 (1.11, 2.04)	1.48 (1.07, 2.04)	1.38 (0.92, 2.05)
Q2 (0.012 - <0.044)	50	5711	0.94 (0.67, 1.33)	0.95 (0.67, 1.36)	0.81 (0.51, 1.30)
Q3 (0.044 - <0.111)	60	5725	1.08 (0.78, 1.50)	1.09 (0.78, 1.52)	0.87 (0.53, 1.43)
Q4 (0.111 - 0.879)	69	5783	1.02 (0.75, 1.39)	1.00 (0.72, 1.38)	0.80 (0.47, 1.34)
P-trend			*0.589*	*0.532*	*0.207*
Duration (years)					
10-year lag					
Unexposed	117	13876	1.00 (reference)	1.00 (reference)	1.00 (reference)
Q1 (1 - <4)	63	7596	0.95 (0.68, 1.32)	0.96 (0.68, 1.37)	1.05 (0.63, 1.76)
Q2 (5 - <9)	79	6531	1.52 (1.11, 2.09)	1.50 (1.08, 2.09)	1.66 (0.99, 2.77)
Q3 (10 - <15)	68	6994	1.15 (0.83, 1.59)	1.16 (0.83, 1.63)	1.32 (0.78, 2.23)
Q4 (16 - 34)	72	6064	1.28 (0.93, 1.76)	1.19 (0.85, 1.66)	1.25 (0.68, 2.30)
P-trend			*0.068*	*0.194*	*0.574*
15-year lag					
Unexposed	126	15259	1.00 (reference)	1.00 (reference)	1.00 (reference)
Q1 (1 - <4)	68	8025	0.96 (0.70, 1.32)	0.98 (0.70, 1.37)	1.00 (0.62, 1.61)
Q2 (5 - <8)	72	5878	1.53 (1.12, 2.10)	1.49 (1.07, 2.07)	1.47 (0.90, 2.42)
Q3 (9 - <13)	55	6045	1.03 (0.73, 1.44)	1.03 (0.72, 1.46)	0.98 (0.58, 1.67)
Q4 (14 - 34)	78	5854	1.17 (0.86, 1.59)	1.13 (0.82, 1.56)	1.03 (0.58, 1.81)
P-trend			*0.237*	*0.378*	*0.953*
20-year lag					
Unexposed	141	18146	1.00 (reference)	1.00 (reference)	1.00 (reference)
Q1 (1 - <3)	62	6787	1.05 (0.76, 1.44)	1.08 (0.77, 1.51)	1.08 (0.70, 1.66)
Q2 (4 - <6)	50	5032	1.16 (0.82, 1.63)	1.16 (0.81, 1.66)	1.12 (0.71, 1.76)
Q3 (7 - <11)	67	5830	1.18 (0.86, 1.62)	1.15 (0.83, 1.59)	1.05 (0.66, 1.65)
Q4 (12 - 34)	79	5265	1.16 (0.85, 1.56)	1.12 (0.82, 1.53)	0.98 (0.59, 1.62)
P-trend			*0.271*	*0.441*	*0.803*

Abbreviations: Q=quartile

[a]Adjusted for age (as the time scale).

[b]Adjusted for age (as the time scale) and smoking.

[c]Adjusted for age (as the time scale), smoking, welding fumes, asbestos and crystalline silica

[d]Missing values were imputed

Lagged analysis by histological subtype

Table S5b. Hazard Ratios (HR) of major histological subtypes according to lagged benzene exposure among males in the Norwegian Offshore Petroleum Workers (NOPW) cohort, 1999–2021.

	**Adenocarcinoma[a]**			**Squamous cell carcinoma [a]**			**Small cell carcinoma [a]**		
**Benzene metric**	**C**	**PYs**	**HR[b] (95% CI)**	**C**	**PYs**	**HR[b] (95% CI)**	**C**	**PYs**	**HR[b] (95% CI)**
Cumulative (ppm-years)									
10-year lag									
Unexposed	48	13010	1.00 (reference)	28	12882	1.00 (reference)	15	12739	1.00 (reference)
Q1 (0.000 - <0.018)	28	6361	1.09 (0.53, 2.24)	18	6211	0.96 (0.38, 2.45)	13	6199	1.01 (0.33, 3.10)
Q2 (0.018 - <0.067)	21	6328	1.00 (0.45, 2.24)	21	6191	0.77 (0.28, 2.10)	12	6169	0.94 (0.29, 3.08)
Q3 (0.067 - <0.159)	33	6354	1.69 (0.75, 3.80)	5	6203	0.18 (0.05, 0.68)	10	6193	0.62 (0.16, 2.46)
Q4 (0.159 - 0.879)	22	6387	0.98 (0.38, 2.52)	16	6232	0.36 (0.09, 1.38)	12	6215	0.99 (0.28, 3.57)
P-trend			*0.621*			*0.205*			*0.730*
15-year lag									
Unexposed	50	14258	1.00 (reference)	30	14095	1.00 (reference)	17	13944	1.00 (reference)
Q1 (0.000 - <0.016)	28	6052	1.13 (0.58, 2.23)	17	5919	0.89 (0.37, 2.14)	11	5889	0.76 (0.26, 2.23)
Q2 (0.016 - <0.058)	19	5996	0.95 (0.43, 2.10)	19	5857	0.72 (0.27, 1.94)	10	5854	0.77 (0.25, 2.36)
Q3 (0.058 - <0.138)	33	6037	1.52 (0.69, 3.31)	6	5894	0.19 (0.05, 0.76)	11	5889	0.67 (0.19, 2.32)
Q4 (0.138 - 0.879)	22	6097	0.88 (0.36, 2.18)	16	5955	0.35 (0.10, 1.30)	13	5938	0.95 (0.30, 2.99)
P-trend			*0.445*			*0.183*			*0.582*
20-year lag									
Unexposed	56	16903	1.00 (reference)	32	16671	1.00 (reference)	18	16514	1.00 (reference)
Q1 (0.000 - <0.013)	28	5351	1.25 (0.67, 2.33)	16	5241	1.08 (0.49, 2.36)	11	5216	0.99 (0.38, 2.60)
Q2 (0.013 - <0.046)	17	5349	0.78 (0.36, 1.67)	15	5211	0.77 (0.31, 1.88)	10	5232	1.04 (0.35, 3.06)
Q3 (0.046 - <0.113)	28	5403	1.23 (0.59, 2.56)	10	5297	0.47 (0.17, 1.34)	8	5344	0.69 (0.22, 2.15)
Q4 (0.113 -0.879)	23	5433	0.87 (0.37, 2.03)	15	5298	0.51 (0.16, 1.55)	15	5208	1.42 (0.55, 3.70)
P-trend			*0.563*			*0.256*			*0.233*
Duration (years)									
10-year lag									
Unexposed	48	13010	1.00 (reference)	28	12882	1.00 (reference)	15	12739	1.00 (reference)
Q1 (1 - <4)	18	7050	0.64 (0.29, 1.41)	12	6981	0.73 (0.24, 2.16)	12	6957	1.06 (0.31, 3.60)
Q2 (5 - <9)	28	6003	1.60 (0.71, 3.57)	18	5849	1.06 (0.40, 2.82)	12	5807	0.99 (0.33, 3.00)
Q3 (10 - <15)	26	6670	1.49 (0.66, 3.38)	13	6481	0.76 (0.27, 2.09)	10	6451	0.83 (0.26, 2.62)
Q4 (16 - 34)	32	5708	1.93 (0.78, 4.79)	17	5526	0.84 (0.22, 3.16)	13	5560	1.00 (0.26, 3.87)
P-trend			0.042			0.842			0.896
15-year lag									
Unexposed	50	14258	1.00 (reference)	30	14095	1.00 (reference)	17	13944	1.00 (reference)
Q1 (1 - <4)	21	7459	0.76 (0.37, 1.56)	13	7374	0.70 (0.26, 1.90)	10	7353	0.71 (0.22, 2.28)
Q2 (5 - <8)	25	5404	1.51 (0.70, 3.29)	15	5261	0.87 (0.32, 2.37)	13	5206	1.11 (0.40, 3.08)
Q3 (9 - <13)	25	5765	1.42 (0.64, 3.13)	11	5585	0.61 (0.22, 1.74)	7	5560	0.52 (0.17, 1.64)
Q4 (14 - 34)	31	5553	1.41 (0.60, 3.29)	19	5404	0.76 (0.23, 2.46)	15	5450	0.98 (0.29, 3.30)
P-trend			0.223			0.702			0.901
20-year lag									
Unexposed	56	16903	1.00 (reference)	32	16671	1.00 (reference)	18	16514	1.00 (reference)
Q1 (1 - <3)	20	6323	0.90 (0.46, 1.76)	13	6250	0.97 (0.41, 2.26)	7	6173	0.71 (0.22, 2.31)
Q2 (4 - <6)	17	4679	1.06 (0.52, 2.19)	9	4525	0.72 (0.27, 1.91)	12	4530	1.52 (0.58, 4.00)
Q3 (7 - <11)	26	5511	1.22 (0.60, 2.50)	14	5379	0.74 (0.30, 1.83)	11	5372	0.94 (0.36, 2.46)
Q4 (12 - 34)	33	5023	1.38 (0.62, 3.06)	20	4894	0.86 (0.32, 2.30)	14	4924	1.11 (0.38, 3.27)
P-trend			0.313			0.737			0.785

Abbreviations: C=Cases, PYs = Person years

[a]Adjusted for age (as the time scale), smoking, welding fumes, asbestos and crystalline silica.

[b]Missing values in covariates were imputed.

Most recent benzene exposure

Table S6. Hazard Ratios (HR) of lung cancer according to most recent benzene exposure among males in the Norwegian Offshore Petroleum Workers (NOPW) cohort, 1999–2021. Based on extrapolated benzene exposure data during follow up among those employed in 1998.

			Model 1[a]	Model 2[b]	Model 3[c]
**Benzene metric**	**Cases**	**Person yrs.**	**HR[d](95% CI)**	**HR[d](95% CI)**	**HR[d](95% CI)**
Cumulative (ppm-years)					
Unexposed	312	30378	1.00 (reference)	1.00 (reference)	1.00 (reference)
Q1 (0.001 - <0.026)	23	2646	0.90 (0.56, 1.45)	0.94 (0.57, 1.55)	0.77 (0.45, 1.31)
Q2 (0.026 - <0.034)	20	2756	0.69 (0.42, 1.13)	0.73 (0.44, 1.23)	0.66 (0.38, 1.15)
Q3 (0.034 - <0.109)	26	2618	1.07 (0.68, 1.67)	0.86 (0.53, 1.39)	0.75 (0.44, 1.27)
Q4 (0.109 - 0.138)	18	2663	0.68 (0.40, 1.15)	0.77 (0.45, 1.34)	0.71 (0.39, 1.26)
P-Trend			0.167	0.251	0.160
Most recent 10 years					
Unexposed	290	27932	1.00 (reference)	1.00 (reference)	1.00 (reference)
Q1 (0.000 - <0.034)	31	3234	1.24 (0.82, 1.88)	1.33 (0.87, 2.04)	1.12 (0.71, 1.76)
Q2 (0.034 - <0.067)	30	3468	0.86 (0.57, 1.30)	0.93 (0.60, 1.44)	0.81 (0.51, 1.30)
Q3 (0.067 - <0.149)	25	3162	0.80 (0.51, 1.25)	0.66 (0.41, 1.05)	0.56 (0.34, 0.92)
Q4 (0.149 - 0.279)	23	3265	0.70 (0.44, 1.11)	0.78 (0.48, 1.26)	0.69 (0.41, 1.18)
P-trend			0.078	0.125	0.060
Most recent 15 years					
Unexposed	261	24834	1.00 (reference)	1.00 (reference)	1.00 (reference)
Q1 (0.000 - <0.043)	39	3961	1.77 (1.24, 2.53)	1.83 (1.28, 2.60)	1.60 (1.10, 2.33)
Q2 (0.043 - <0.097)	31	4091	0.88 (0.59, 1.32)	0.88 (0.58, 1.33)	0.73 (0.46, 1.14)
Q3 (0.097 - <0.180)	41	4093	0.95 (0.66, 1.37)	0.87 (0.59, 1.28)	0.76 (0.49, 1.18)
Q4 (0.180 - 0.441)	27	4080	0.68 (0.44, 1.04)	0.73 (0.47, 1.14)	0.64 (0.39, 1.06)
P-trend			0.050	0.097	0.045
Intensity (ppm/years)					
Most recent 5 years					
Unexposed	312	30378	1.00 (reference)	1.00 (reference)	1.00 (reference)
Q1 (0.001 - <0.007)	29	3268	0.88 (0.57, 1.35)	0.93 (0.59, 1.47)	0.78 (0.48, 1.28)
Q2 (0.007 - <0.010)	18	2080	0.87 (0.51, 1.47)	0.85 (0.49, 1.50)	0.73 (0.40, 1.31)
Q3 (0.010 - <0.027)	20	2634	0.89 (0.54, 1.48)	0.73 (0.43, 1.25)	0.66 (0.38, 1.17)
Q4 (0.027 - 0.041)	20	2700	0.68 (0.42, 1.12)	0.78 (0.47, 1.30)	0.70 (0.41, 1.22)
P-trend			0.118	0.203	0.131
Most recent 10 years					
Unexposed	290	27932	1.00 (reference)	1.00 (reference)	1.00 (reference)
Q1 (0.000 - <0.007)	34	3291	1.22 (0.82, 1.82)	1.32 (0.86, 2.02)	1.10 (0.69, 1.74)
Q2 (0.007 - <0.009)	23	3255	0.67 (0.42, 1.06)	0.68 (0.42, 1.11)	0.58 (0.35, 0.98)
Q3 (0.009 - <0.026)	28	3277	1.04 (0.68, 1.60)	0.85 (0.54, 1.34)	0.76 (0.47, 1.24)
Q4 (0.026 - 0.046)	24	3305	0.69 (0.44, 1.09)	0.79 (0.49, 1.26)	0.72 (0.43, 1.20)
			0.104	0.188	0.095
Most recent 15 years					
Unexposed	261	24834	1.00 (reference)	1.00 (reference)	1.00 (reference)
Q1 (0.000 - <0.007)	42	4037	1.41 (0.98, 2.03)	1.46 (0.99, 2.15)	1.21 (0.79, 1.87)
Q2 (0.007 - <0.008)	33	4055	0.88 (0.60, 1.31)	0.97 (0.64, 1.45)	0.89 (0.57, 1.38)
Q3 (0.008 - <0.021)	33	4045	1.01 (0.69, 1.50)	0.81 (0.53, 1.23)	0.69 (0.43, 1.11)
Q4 (0.021 - 0.049)	30	4089	0.76 (0.51, 1.14)	0.84 (0.55, 1.28)	0.75 (0.47, 1.22)
P-trend			0.184	0.250	0.110

[a]Adjusted for age (as the time scale).

[b]Adjusted for age (as the time scale), smoking

[c]Adjusted for age (as the time scale), smoking, welding fumes, asbestos and crystalline silica (not extrapolated)

[d]Missing values in covariates were imputed

Time-varying benzene exposure during follow-up

**Table S7.** Hazard Ratios (HR) of lung cancer according to time-varying benzene exposure among 25,347 males in the Norwegian Offshore Petroleum Workers (NOPW) cohort, 1999–2021. Based on extrapolated data during follow up among those employed in 1998.					
			**Model 1[a]**	**Model 2[b]**	**Model 3[c]**
**Benzene metric**	**Cases**	**Person years**	**HR[d](95% CI)**	**HR[d](95% CI)**	**HR[d](95% CI)**
Cumulative (ppm-years)					
Unexposed	112	13034	1.00 (reference)	1.00 (reference)	1.00 (reference)
Q1 (0.000 - <0.021)	82	7012	1.40 (1.02, 1.91)	1.34 (0.96, 1.87)	1.48 (0.91, 2.42)
Q2 (0.021 - <0.091)	65	6952	1.32 (0.94, 1.83)	1.34 (0.94, 1.90)	1.40 (0.78, 2.53)
Q3 (0.091 - <0.234)	67	7018	1.13 (0.81, 1.56)	1.14 (0.81, 1.60)	1.20 (0.68, 2.12)
Q4 (0.234-1.289)	73	7045	1.10 (0.80, 1.51)	1.05 (0.75, 1.46)	1.03 (0.57, 1.86)
P-Trend			0.727	0.551	0.196
**Duration (years)**					
Unexposed	112	13034	1.00 (reference)	1.00 (reference)	1.00 (reference)
Q1 (1 - <5)	74	8166	1.10 (0.80, 1.51)	1.13 (0.81, 1.59)	1.33 (0.79, 2.25)
Q2 (6 - <12)	77	6365	1.71 (1.25, 2.36)	1.58 (1.12, 2.21)	1.66 (0.97, 2.83)
Q3 (13 - <22)	60	6647	1.33 (0.95, 1.85)	1.25 (0.89, 1.77)	1.32 (0.77, 2.28)
Q4 (23-50)	76	6848	0.99 (0.72, 1.35)	0.99 (0.71, 1.37)	0.98 (0.56, 1.73)
P-trend			0.984	0.909	0.169
[a]Adjusted for age (as the time scale).					
[b]Adjusted for age (as the time scale), smoking					
[c]Adjusted for age (as the time scale), smoking, welding fumes, asbestos and crystalline silica					
[d]Missing values in covariates were imputed					

Lung cancer risk according to employment duration

Table S8. Hazard ratios (HR) with 95% confidence intervals (CIs) of lung cancer according to employment duration among males in the Norwegian Offshore Petroleum Workers cohort, 1999–2021

Cancer site	Employment duration	Cases/Non Cases	HR[a] (95% CI)
All lung (n= 399)			
	Q1 (0 - 4)	81/435	1.00 (reference)
	Q2 (5 - 10)	90/456	1.03 (0.72, 1.47)
	Q3 (11 - 14)	65/338	1.09 (0.74, 1.60)
	Q4 (15 - 19)	84/402	0.97 (0.67, 1.40)
	Q5 (20 - 34)	79/404	0.95 (0.66, 1.37)
	P-Trend		*0.72*
Adenocarcinoma (n= 152)			
	Q1 (0 - 4)	30/441	1.00 (reference)
	Q2 (5 - 10)	27/467	0.85 (0.49, 1.49)
	Q3 (11 - 15)	30/424	1.17 (0.68, 2.03)
	Q4 (16 - 20)	41/440	1.29 (0.76, 2.18)
	Q5 (21 - 34)	24/311	1.26 (0.69, 2.29)
	P-Trend		*0.183*
Squamous cell carcinoma (n=88)			
	Q1 (0 - 4)	19/443	1.00 (reference)
	Q2 (5 - 10)	24/465	1.19 (0.61, 2.30)
	Q3 (11 - 14)	13/351	0.91 (0.43, 1.94)
	Q4 (15 - 19)	19/414	0.89 (0.44, 1.76)
	Q5 (20 - 34)	13/416	0.61 (0.28, 1.31)
	P-Trend		*0.127*
Small cell carcinoma (n=62)			
	Q1 (0 - 4)	13/445	1.00 (reference)
	Q2 (5 - 10)	11/469	0.78 (0.34, 1.84)
	Q3 (11 - 14)	13/351	1.32 (0.57, 3.02)
	Q4 (15 - 20)	13/524	0.71 (0.32, 1.62)
	Q5 (21 - 34)	12/315	1.13 (0.51, 2.52)
	P-Trend		*0.972*

Abbreviations: Q = Quartile

[a]adjusted for age, smoking, and education.

**Table S9**. Spearman rank correlation Coefficients (r_{sp}) for occupational co-exposures						
**Metric**	**Exposure**	*Benzene*	*Crystalline silica*	*Diesel exhaust*	*Welding fumes*	*Asbestos*
**Cumulative (ppm-years)**	*Benzene*	1				
	Crystalline silica	0.0376	1			
	Diesel exhaust	0.4059	0.3451	1		
	Welding fumes	0.6608	0.1256	0.4020	1	
	Asbestos	0.6324	0.5119	0.5211	0.3949	1
**Duration (years)**	*Benzene*	1				
	Crystalline silica	0.2420	1			
	Diesel exhaust	0.5135	0.4430	1		
	Welding fumes	0.6360	0.2649	0.5268	1	
	Asbestos	0.8026	0.4758	0.4285	0.4745	1
**Average intensity (ppm)**	*Benzene*	1				
	Crystalline silica	-0.0504	1			
	Diesel exhaust	0.3318	0.3246	1		
	Welding fumes	0.6175	0.0283	0.3134	1	
	Asbestos	0.4356	0.5037	0.5499	0.3110	1
